# Daughter's college completion and parents' psychosocial wellbeing: quasi-experimental evidence from South Korea

**DOI:** 10.1016/j.ssmph.2025.101846

**Published:** 2025-07-23

**Authors:** Ah-Reum Lee, K. Renata Flores Romero, Jacqueline Torres

**Affiliations:** Department of Epidemiology and Biostatistics, University of California, San Francisco, USA

**Keywords:** Education, Population aging, Intergenerational effects, Quasi-experimental methods, South Korea

## Abstract

The educational achievements of offspring are reported to be positively associated with the wellbeing of older parents, although observational research on this topic is likely subject to residual confounding bias. We use data on over 7,000 older adults who responded to the Korean Longitudinal Study on Aging (KLoSA), a population-based study that captures data on the wellbeing and family dynamics of adults 45 years along with detailed rosters of respondents' living children, including their age and educational attainment. We use these rosters to characterize offspring exposure to a 1993 higher education reform that increased college enrollment rates, particularly for young women, by over 45 percentage points over a decade. Prior research has leveraged this reform to identify the causal effects of college completion on women's fertility and labor market outcomes; we similarly demonstrate its utility as a natural experiment in our study, showing a meaningful discontinuity in the college completion of respondents' daughters based on exposure to the reform. We show that college completion among daughters reduced the risk of depression for older parents by 6–25% (with variation by parents' gender and model specification) but had no effects on ratings of life satisfaction. The returns to offspring college completion were most pronounced for older mothers, parents in living urban regions and those with fewer of their own household assets. Daughter's college completion also had an impact on some domains of intergenerational support that could explain effects on depressive symptoms, including the frequency of parent-child contact.

## Highlights

• First quasi-experimental study on offspring's college and parental well-being.
• Offspring's college completion reduces parents' depressive symptoms.
• Stronger effects for mothers, urban parents, and lower-asset households.
• College fosters upward intergenerational spillover, especially via daughters.
• Educational mobility enhances parents' well-being, especially for the disadvantaged.

## 1 Introduction

Educational attainment is an important determinant of later-life depressive symptoms (Powdthavee et al., 2015; Cohen et al., 2020; Richardson et al., 2020), which are in turn associated with quality of life (Gertner et al., 2017; Gilman et al., 2017) and the risk of chronic disease and mortality (Holwerda et al., 2016; Machado et al., 2018; Naicker et al., 2017; Pratt et al., 2016; Russ et al., 2012). The mental health benefits of education may also be transferable to others in the family network. Drawing on the concept of “linked lives” – the idea that close family members' experiences and life outcomes are interconnected (Elder, 1998; Gilligan et al., 2018; Greenfield & Marks, 2006) – recent research suggests that adult children's higher SES, including educational attainment, can benefit their parents' psychological well-being through “upward” spillover effects (De Neve & Kawachi, 2017).

There are multiple pathways through which a child's education can influence parents' mental health. In South Korea, where public support for older adults is limited and parents often shoulder the financial cost of education, having financially secure children can reduce parental stress and burden (Kim & Cook, 2011; Ryff et al., 1994). In China, similar mechanisms operate, with studies linking children's education to improved parental cognitive functioning, partly through financial transfers (Ma, 2019). Culturally, education also provides symbolic value: in societies where filial piety is emphasized, such as Korea, parents may feel a sense of pride and enhanced social identity from their children's educational achievements (Swartz et al., 2011; Park, 2018). Moreover, higher-educated children tend to provide health information and support, improving parents' care-seeking and daily health management (Thoits, 2011). This support becomes critical in the context of an aging population and rising healthcare pressures (OECD, 2020). Finally, emotional and relational benefits, such as improved intergenerational ties, also contribute to better psychological outcomes for parents (Xu & Luo, 2022).

A growing body of empirical research suggests that adult children's educational attainment is positively associated with their aging parents' psychosocial well-being, independent of the parents' own education levels. This relationship has been documented across diverse global contexts, including Europe, Mexico, Taiwan, and the United States (Sabater & Graham, 2016; Lee et al., 2017; Lee C., 2018; Yahirun et al., 2020; Torres et al., 2022; Gutierrez et al., 2024). However, much of this evidence stems from observational studies, which are susceptible to confounding. Unmeasured factors, such as socioeconomic background, early-life health conditions, personality traits, and living environments, may jointly shape both children's educational outcomes and parents' mental health, complicating efforts to draw causal conclusions.

The most rigorous studies on this topic have used quasi-experimental designs to better identify causal effects, reducing concerns about unmeasured confounding bias (Gutierrez et al., 2024; Ma, 2019; Torres et al., 2022). However, these studies have concentrated on the effects of increased offspring education towards the lower end of the education distribution (e.g., increases to lower or upper secondary schooling). Estimates of the causal effects of offspring college education may be more applicable to many global settings in which mandatory minimum levels of schooling already exceed secondary schooling and policy debates are centered around the value of higher education (OECD Reviews of Pension Systems: Korea, 2022).

This research gap is particularly salient in contexts like South Korea, where rapid aging and insufficient public support for the elderly place significant caregiving and financial burdens on families (Rhee et al., 2015). Despite having a national pension system (NPS), many older adults receive low payouts, and the absence of a senior-specific public health insurance program exacerbates their vulnerability (OECD Reviews of Pension Systems: Korea, 2022). At the same time, South Korea has witnessed a significant expansion in higher education over recent decades (Choi, 1996; Choi & Lee, 2017). In light of these structural challenges, higher educational attainment among adult children may be an increasingly important private resource – one that not only shapes life chances but may also serve as a buffer for aging parents facing late-life health and financial insecurity.

We provide the first-ever quasi-experimental study of the impact of offspring college education on older parents’ late-life outcomes. We leverage a 1993 higher education reform in South Korea as a natural experiment (Choi, 1996; Choi & Lee, 2017). This reform marked a significant turning point that occurred when a newly elected government eased college admission restrictions by removing fixed student quotas per higher education institution, thereby expanding access. This reform resulted in a dramatic increase in college enrollment rates, rising by over 45 percentage points between 1993 and 2004 (Kim & Lee, 2006; Oh, 2011), with evidence of causal impacts on labor market outcomes (Sohn & Lee, 2019) as well as earnings (Choi, 2015) for the cohorts who directly benefitted (See SI Appendix 1 for the institutional background of the reform).

We use population-based data from the Korean Longitudinal Study on Aging (KLoSA), including 7093 adults 51 years and older who responded during survey years fielded between 2006 and 2020 (Korea Employment Information Service, 2025). KLoSA collected detailed data on the age and educational attainment of each of the respondents' living children, in addition to sociodemographic and health on respondents themselves. We used adult children's exposure to the higher education reform based on their calculated birth year as an instrumental variable (IV) which, under standard assumptions for IV (Angrist, Imbens, & Rubin, 1996), allows us to identify the local average treatment effect of offspring college completion on the depressive symptoms of older parents. KLoSA respondents reported their depressive symptoms in the past two weeks via the 10-item Center for Epidemiologic Studies Depression Scale (CES-D 10; range 0–30 points) (Radloff, 1977; Irwin et al., 1999). The CES-D 10 was translated into Korean, and the reliability and validity of the Korean version have been established (Cho and Kim, 1998; Kang et al., 2024).

Drawing on the social determinants of health framework, which emphasizes the role of income, education, and employment in shaping health outcomes (Braveman & Gottlieb, 2014), this study extends prior research on the intergenerational effects of adult children's socioeconomic status (Gutierrez et al., 2024; Lee, 2018; Lee et al., 2017; Ma, 2019). We hypothesize that higher educational attainment in children is associated with improved psychosocial well-being among parents in South Korea. This influence is theorized to occur through several pathways, including enhanced intergenerational financial transfers, emotional closeness, and parental satisfaction with children's achievements. The rich questions about financial transfers and other connections to adult children in KLoSA allow us to empirically examine these potential mechanisms in our quasi-experimental framework. Specifically, we consider secondary measures of intergenerational contact and support as well as of life satisfaction and satisfaction with adult children, with measures detailed in SI Appendix 2. Additionally, we consider heterogeneous returns to offspring college completion by parent sex/gender and socioeconomic status given some evidence from other settings of differential effects across population subgroups (Sabater et al., 2020).

Finally, this research pays particular attention to the role of child gender. We specifically examine the impact of increased educational attainment among daughters, given the country's traditional gender norms around caregiving for aging parents, alongside the observed decline in adherence to such roles. In Korean culture, caregiving responsibilities have historically been shaped by a patrilineal family system that places greater investments and expectations on sons, particularly the eldest, who are often expected to serve as the primary caregivers for aging parents (Kim et al., 2015; Lee et al., 1994). In addition, coresidence with adult sons is more common for older Koreans (Chung, 2018), such that increased educational attainment among sons could be more impactful for older parents' depressive symptoms. Alternatively, studies from across global settings suggest that daughters frequently deliver more attentive and responsive care (Bott et al., 2017; Poisson et al., 2023), which is essential for parents coping with depression or psychosocial challenges – a dynamic that reflects the shifting caregiving responsibilities shaped by traditional gender norms. Importantly, prior research has shown that women were more likely to benefit from the 1993 reform (Ahn, 2011), such that the educational, labor market, and earnings gains were more substantial for young women (Sohn & Lee, 2019). As more daughters pursue higher education, participate in the workforce, and expand their caregiving roles, their socioeconomic accomplishments have the potential to greatly enhance the psychosocial well-being of their elderly parents. In light of evolving gender roles and economic contributions, the influence of a daughter's educational level on the health and well-being of older parents in South Korea warrants a focused investigation.

## 2 Data and methods

**Study Design and Participants**. This study utilized data from the Korean Longitudinal Study of Aging (KLoSA), a nationally representative biannual panel survey of individuals aged 45 and above. The baseline sample, collected in 2006, used a stratified, multi-stage area probability design and consisted of 10,254 individuals. In 2014, a refreshment sample of 920 individuals born in 1962 or 1963 was added. The survey followed respondents until the end of 2020, resulting in eight waves over sixteen years. Our analytic sample was drawn from 11,174 respondents interviewed between 2006 and 2020.

We employed several restrictive criteria for two scenarios: one where the index child is the oldest, and another where the index child is the most highly educated in the family (see “**Adult Child Education**” below for further details on the index child). First, we excluded respondents under the age of 51 (n = 481) due to the relatively few participants who had children aged 25 or older in this age group. Additionally, the use of this age threshold aligns with prior studies in the literature, which commonly apply similar criteria to focus on older adults. We further eliminated those whose child was aged 25 or younger at baseline, in order to focus on offspring who had likely completed their schooling (excluding n = 299 for the analyses focused on oldest children; excluding n = 301 for analyses focused on the highest-educated child). Additionally, we excluded respondents whose children were not part of the birth cohort affected by the reform (born between 1974 and 1983; treatment group) or the preceding 10-year cohort (born between 1964 and 1973; control group) (n = 2,934 for the oldest child; n = 3,331 for the highest-educated child). We also excluded individuals with missing data on the outcome measures and covariates (n = 367 for the oldest child; n = 354 for the highest-educated child). Additionally, we tested for missingness and found the data were not missing completely at random. However, multiple imputation using chained equations produced results similar to the complete-case analysis (see SI Appendix 3) (Azur et al., 2011). We therefore proceeded with the complete-case approach and excluded observations with missing data. After applying these criteria, the final analytic sample consisted of 7,093 respondents with the oldest child (4,001 women and 3,092 men), and 6,707 respondents (3,792 women and 2,915 men) with the highest-educated child.

**Fig. 1:**
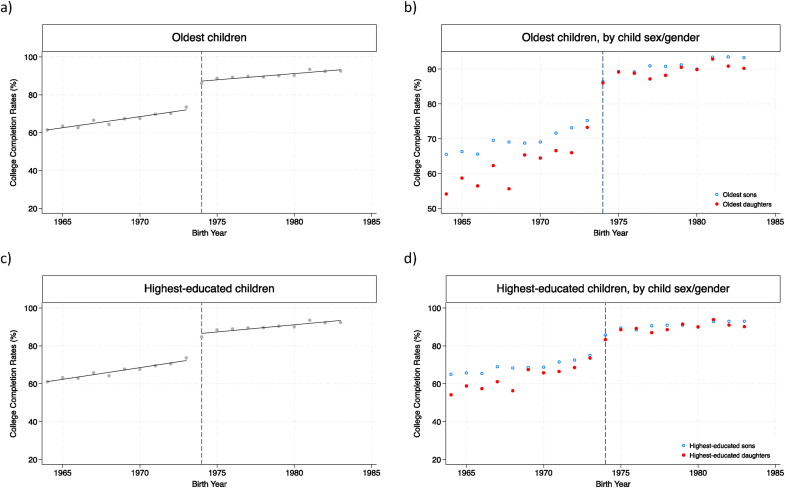
College Completion Rates by Index Child Birth Cohort, Overall and by Child Sex/Gender Notes: The figures show college completion rates of the oldest and the most highly educated children on either side of the 1974 cutoff, the first cohort affected by the 1993 education reform. See Table S2 for the first-stage estimates of the two-stage least squares (2SLS) IV estimator, which predicts the probability of college completion for the index child based on their exposure to the 1993 reform.

**Instrumental Variable.** We used children's exposure to the Korean 1993 higher education reform as the instrument for offspring educational attainment. This instrument allows us to identify the effects of children's education on parents' psychosocial well-being by isolating the exogenous variation in educational attainment that is independent of family background or inherited characteristics. The 1993 reform expanded access to higher education, impacting those born in 1974 or later (Fig. 1). The variable was assigned a value of 1 if the index child of the respondent was born in or after 1974 (within the 10-year period from 1974 to 1983, representing the treatment group) and was affected by the policy reform. It was assigned a value of 0 if the child was born before 1974 (within the preceding 10-year period, representing the control group).

**Adult Child Education (Exposure)**. Since most families in this analysis have multiple offspring, there are various ways to measure and parameterize the educational attainment of these offspring within the family. Our primary exposure is college completion by an index child (i.e., the highest-educated child or the oldest child), a methodology consistent with previous research (see SI Appendix 4 on the choice of the index child in literature). We present results for both the highest-educated child and the oldest child within our primary analysis. Additionally, we examine years of schooling as a secondary exposure to capture a broader spectrum of educational attainment. For more discussion of the choice of the index child in research on offspring education and parents’ health, see SI Appendix 4.

**Outcomes**. We utilize an outcome-wide longitudinal design to evaluate the effect of a single exposure at a specific time point (i.e., offspring educational attainment) on multiple outcomes that are temporally subsequent to the exposure (i.e., parents’ psychosocial wellbeing). This approach could offer advantages over traditional single exposure-outcome studies, such as reduced investigator bias, higher likelihood of reporting null effects, and better capacity to compare effect sizes (VanderWeele et al., 2020). We employ inverse probability weights (i.e., inverse of the number of observations for that unit) with clustered standard errors to account for the multiple outcome assessments per respondent (Cameron & Miller, 2015; Hernán & Robins, 2020). Data management and statistical analyses were conducted using Stata 18 SE (StataCorp, 2023).

Our primary outcomes were depressive symptoms measured using the 10-item Center for Epidemiologic Studies Depression Scale (CES-D 10) scores (Radloff, 1977). In addition to the continuous scale, we created a binary indicator to identify individuals at risk of depression, employing a cut-off score of 10 (Andresen et al., 1994) to contextualize our findings and aid in interpreting our results. As a sensitivity analysis, we also tested an alternative cut-off of 15 (more commonly used in clinical and psychiatric samples) for elevated risk of depression (Björgvinsson et al., 2013). Our secondary outcomes included intergenerational support from adult children to parents, such as the frequency of contact and financial support, as well as two measures of life satisfaction: overall life satisfaction and satisfaction with relationships with their children. For a detailed description of the outcome measures, see SI Appendix 2.

**Covariates**. The control variables include a range of demographic, socioeconomic, and family-level characteristics of the respondents. Specifically, we accounted for the respondents' characteristics, including age (measured in years), sex/gender (male/female), marital status (married or partnered versus separated, divorced, widowed, or single), educational attainment (measured in years), and birth cohorts; family-level characteristics, including the total number of living children, children's gender composition, whether the respondent's father or mother is alive, household income, household assets, their urban or rural residence, and regions (17 provinces and special cities); current health behaviors (smoking, drinking, vigorous exercise); and early life characteristics such as parental education. Further details on the confounder variables are also included in the SI Appendix 2.

**Statistical Analysis**. To investigate the effects of children's schooling on the psychosocial wellbeing of older parents, we employed two-stage least squares (2SLS) estimation with an instrumental variable (IV). To ensure that the 1993 higher education reform is a valid instrument, we relied on the key identifying assumptions, including the relevance of our instrument (the education reform) affecting the primary exposure (education of the adult child), the exclusion restriction (the reform is correlated with parental psychosocial well-being only through its effect on the adult child's schooling), and independence (there are no unmeasured confounders between the reform and parental well-being). Additionally, we assumed monotonicity, which posits that no individuals would choose a lower level of education after the expansion than the level they chose or would have chosen before the reform (Labrecque & Swanson, 2018). Additional details and justification for these assumptions are provided in SI Appendix 5.

For the first-stage estimation, we applied a linear model to estimate the college completion status by the index child, regressing this on the instrumental variable and covariates. In the second-stage model, we estimated the respondents' CES-D scores using the predicted educational attainment of their index child, which was derived from the first-stage regression. This estimation also incorporated the same set of covariates used in the first stage, in addition to potential mediator variables, including current intergenerational relationships and support, as well as the parents' health behaviors. As part of model diagnostics, we tested for nonlinearity in age and other key covariates by comparing linear, quadratic, and cubic specifications. Although the quadratic specification of age showed a very small improvement in fit (Δ AIC ≈ 10; Δ Adj. R^2^ = 0.001), higher-order terms were not substantively meaningful, and residuals showed no evidence of nonlinearity. For parsimony and efficiency in the 2SLS models, we retained linear terms. We also estimated models stratified by the parent (respondent's) gender and socioeconomic characteristics, including educational attainment, household assets, and urban locality.

For a robustness check, we first tested alternative instrument specifications. This included randomly selecting a child from each household as the index child, rather than consistently choosing the oldest or the most educated. This approach helps to eliminate the possibility that our observed impacts are merely due to the selection of a specific child. Additionally, we used the proportion of children who completed college and the average years of schooling for all children aged 25 and older to capture the broader impact of increased education on the respondents' adult children. We instrumented the proportion of children who completed college with the proportion of children exposed to the 1993 higher education reform, and we used the average years of required schooling as an instrument for the average years of schooling of all children aged 25 and older within households. Second, we examined the effects across different birth cohort bounds (5 and 10 years) to assess how the choice of different birth cohorts and sample size variations influence our estimates. Lastly, we assessed the impact of increased offspring education on parents' psychosocial well-being by categorizing postsecondary institutions in Korea into two groups: 2-year/junior colleges and 4-year universities. The 2-year/junior colleges are often considered lower-tier or alternatives to 4-year colleges in South Korea and, therefore, may not provide the same level of prestige or expected outcomes in terms of graduates’ job prospects and the potential intergenerational spillover (Jung & Lee, 2016; Lee & Vignoles, 2022). Therefore, we aim to shed light on the potential impact of educational expansion by examining not only the increased access to higher education but also the relative quality of the institutions involved.

## 3 Results

**Descriptive Statistics**. Table S1 presents descriptive statistics for older parents in our analytic sample, categorized into the treatment group (parents whose oldest child was born between 1974 and 1983; n = 2,525) and the control group (parents whose oldest child was born between 1964 and 1973; n = 4,568). Compared to the control group, parents in the treatment group are generally younger, more likely to have completed high school education, and live in urban areas. Parents in the treatment group report having fewer children, but their children have a higher percentage of college degrees (80% vs. 47% for the control group), which is expected given their exposure to the 1993 reform. These parents also report fewer depressive symptoms, greater life satisfaction, and more frequent in-person and overall contact with their children. Financial transfers from children are less common in the treatment group, whereas non-financial support is similar across both groups.

**First-stage Results**. We used two-stage least squares (2SLS) estimation to evaluate the effects of offspring educational attainment on parents' psychosocial measures and intergenerational contact and support. Our outcomes of interest and the covariates are described in detail in SI Appendix 2. First, we tested the relevance of the 1993 reform for the educational attainment of adult children of KLoSA respondents (i.e., the first stage of 2SLS IV estimator). We found that exposure to the reform was associated with a 14.6 percentage point (pp) increase in college completion rates (95% CI: 10.70, 18.54) and an increase of 0.5 years (95% CI: 0.36, 0.73) in average years of schooling among the oldest children of KLoSA respondents (Fig. 1, Table S2). The magnitude of the first-stage estimate was particularly large for oldest daughters: exposure to the reform among oldest daughters was associated with a 19.4-pp increase in college completion rates (95% CI: 13.59, 25.30) and 0.8 years of additional schooling (95% CI: 0.53, 1.06). Partial F-statistics (F = 53.4 for the oldest children overall and F = 42.4 for the oldest daughters) supported the strength of using exposure to the 1993 reform as an instrumental variable. The magnitude of the first-stage estimate was smaller but still significant for the oldest sons, with a 10.6-pp increase (95% CI: 5.37, 15.48) in college completion rates and an increase of 0.33 years (95% CI: 0.06, 0.59) in average years of schooling. However, the partial F-statistic for the oldest sons' average years of schooling (F = 6.0) was below the standard threshold of 10, while the F-statistic for college completion rates just crossed the threshold (F = 15.8) (Staiger & Stock, 1997). These patterns could reflect the fact that the 1993 reform was most beneficial for expanding access to higher education for young women, which has been suggested by other scholars (Ahn, 2011). Given the significant impact of the 1993 education reform on daughters’ education, as indicated by high partial F-statistics, the rest of our analysis focuses on assessing how daughters' college completion affects their parents' psychosocial well-being. Patterns were generally similar when we calculated first-stage estimates when instead selecting the highest-educated child or a randomly selected child (vs. the oldest child) as the “index” child for determining exposure, although the magnitude of first-stage estimates was smaller.

**Fig. 2:**
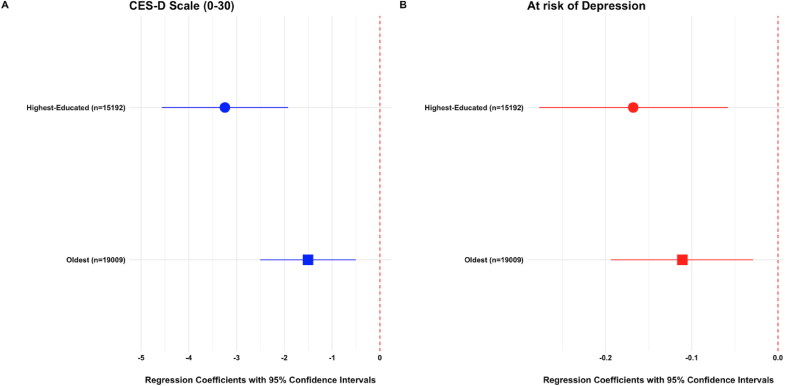
Beta Coefficients and 95% Confidence Intervals from Two-Stage Least Squares (2SLS) Regression for Predicting Parents' Depressive Symptoms and Risk of Depression Based on Daughters' Education, by Type of Index Child (Highest-educated vs. Oldest) Notes: The CES-D 10 scale measures depressive symptoms over the past two weeks, with scores ranging from 0 to 30, with higher scores indicating a greater frequency of negative feelings over the past two weeks. The risk of depression is defined as having a score above the cut-off of 10 on the CES-D 10 scale. See Table S3 notes for model specification.

**Impact of Daughter's Education on Parents' Psychosocial Well-Being**. Among KLoSA respondents, an increased probability of college completion among their oldest daughter was associated with fewer depressive symptoms (Fig. 2; Table S3). Each percentage-point increase in the probability of the oldest daughter completing college corresponded to a 1.5-point decrease in the CES-D score (β = -1.51, 95% CI: -2.51, -0.50). The increase in the probability of the highest educated daughter completing college was associated with an even larger decrease in CES-D score at 3.2 points (β = -3.25, 95% CI: -4.57, -1,92). These reductions are close to the value of one depressive symptom, which is quantified as three points on the 30-point 10-item CES-D scale. Findings were consistent when instead modeling results as a function of years of offspring schooling rather than the probability of college completion: each additional year of schooling for the oldest daughter was associated with a 0.7-point decrease in the CES-D score (β = -0.74, 95% CI: -0.98, -0.49), and each additional year for the most highly educated daughter corresponded to a 0.9-point decrease (β = -0.88, 95% CI: -1.24, -0.51).

Results followed similar patterns when using binary indicators at both the conventional (CES-D ≥10) and elevated (CES-D ≥15) thresholds (Fig. 2; Table S3), although the significance of the association varied depending on the selection of the index child (the oldest child vs. most educated child). For example, each percentage-point increase in the probability of the highest-educated daughter completing college was associated with a 16.8-pp decrease in the likelihood of being at risk of depression (CES-D ≥ 10) (β = -0.17, 95% CI: -0.28, -0.06), and a 7.2-pp decrease in the likelihood of being at elevated risk (CES-D ≥ 15) (β = -0.07, 95% CI: -0.14, -0.01). In contrast, the association was weaker when using the oldest daughter as the index child: a corresponding increase in her college completion probability was linked to an 11.1-pp reduction in depression risk (β = -0.11, 95% CI: -0.19, 0.03), but no significant change at the elevated-risk threshold (β = 0.00, 95% CI: -0.05, 0.05). Similar trends were observed when modeling years of schooling instead of college completion.

**Evaluating heterogeneity**. We further evaluated heterogeneity by parents' sex/gender and socioeconomic characteristics, including their educational attainment (less than high school vs. high school graduates or above), household assets (below and above median), and urban locality (rural vs. urban). However, partial F-statistics were weak (below 10), and the 95% confidence intervals were wide for specific subgroups when stratified by parents’ socioeconomic characteristics, suggesting weaker instruments for these groups, which could lead to biased estimates. This was particularly evident among parents who have a high school education or higher (relevant for both subgroups with the oldest daughters and those with the highest-educated daughters as an index child), as well as among parents residing in urban areas with the highest-educated daughters as an index child. Due to these weak instruments, we limited second-stage analyses to subgroups with sufficiently robust first-stage estimates.

**Fig. 3:**
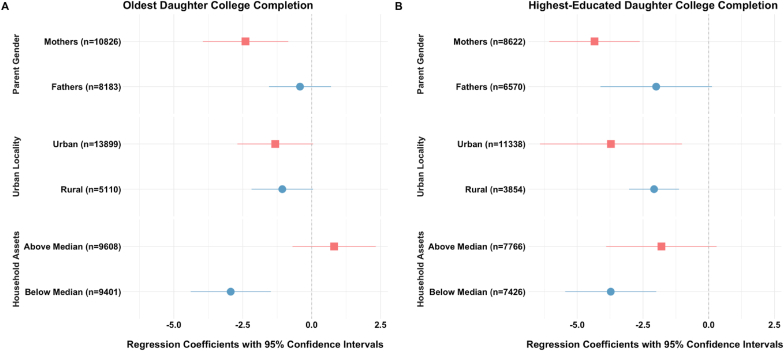
Beta Coefficients and 95% Confidence Intervals from Two-Stage Least Squares (2SLS) Regression for Predicting Parents' Depressive Symptoms Based on Daughters' Education, By Parents' Sex/Gender and Socioeconomic Characteristics Notes: 1) The CES-D 10 scale measures depressive symptoms over the past two weeks, with scores ranging from 0 to 30, with higher scores indicating a greater frequency of negative feelings over the past two weeks. See Table S4 and Table S5 notes for model specification. 2) Due to the weak instrument (Partial F = 11.8) in the first stage when using the highest-educated daughter as an index for urban parents, conclusive assessments cannot be made for this subgroup.

In the stratified analysis, estimated associations varied significantly by parents’ gender and the selection of the index child (Fig. 3; Table S4). For instance, each percentage-point increase in the probability of the oldest daughter completing college was associated with a significant decrease in depressive symptoms for mothers (β = -2.40, 95% CI: -3.95, -0.85) but was insignificant for fathers (β = -0.42, 95% CI: -1.55, 0.70). Similarly, the negative association between the highest-educated daughter completing college and depressive symptoms was strong for mothers (β = -4.34, 95% CI: -6.06, -2.62) but relatively weaker for fathers (β = -2.00, 95% CI: -4.12, 0.12). These patterns were also observed when modeling the effects of increased years of schooling.

We further examined binary CES-D thresholds for elevated depression risk (≥10 and ≥15). At the conventional cutoff of 10, oldest daughters’ college completion was significantly associated with lower odds of parental depression, particularly among mothers (β = -0.19, 95% CI: -0.30 0.08). At the more stringent threshold of 15, often used in clinical and psychiatric samples, only the association for mothers remained statistically significant (β = -0.06, 95% CI: -0.12 0.00), while no significant effect was observed for fathers (β = -0.03, 95% CI: -0.05, 0.01). Similar patterns were found when considering the highest-educated daughters.

The decrease in depressive symptoms was more pronounced among urban-dwelling parents (β = -1.32, 95% CI: -2.70, 0.05) compared to rural parents (β = -1.07, 95% CI: -2.18, 0.05) with each percentage-point increase in their eldest daughter's college completion (Fig. 3; Table S5). Similarly, the associations were stronger for urban-dwelling parents (β = -3.71, 95% CI: -6.42, -1.02) compared to those in rural areas (β = -2.08, 95% CI: -3.03, -1.13) with their highest-educated daughters' college completion. However, the wide 95% confidence intervals for urban-dwelling parents indicate considerable variability in this association.

Parents below the median level of household assets exhibited a more substantial decrease in depressive symptoms associated with their oldest daughter's college completion (β = -2.94, 95% CI: -4.39, -1.48) compared to those above the median (β = 0.81, 95% CI: -0.70, 2.33). The associations were generally larger in models highest-educated daughters' college completion, but the 95% confidence intervals overlapped significantly across various model specifications (Fig. 3; Table S5). For parents' own education, comparisons are limited due to weak instruments; hence, we cannot make assessments of heterogeneity.

**Fig. 4:**
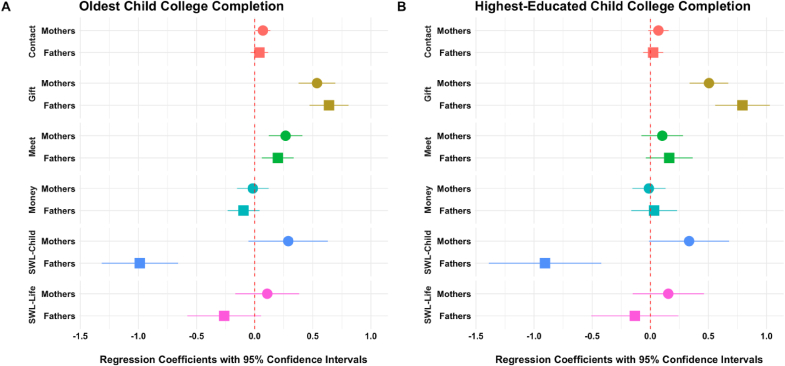
Beta Coefficients and 95% Confidence Intervals from 2SLS Regression for Predicting Intergenerational Support from and Relationships with Daughters and Parents' Life Satisfaction, by Parents' Sex/Gender Notes: We defined a binary variable, assigning a value of 1 to those parents who had contact with their children more than once a month (“contact”), who received a non-financial gift from children in the last year (“gift”), who met their children once a month (“meet”), or who received financial support from their children in the last year (“money”). Parent's life satisfaction was recoded as standardized z-scores. Higher scores indicate satisfaction with the relationship with their daughter (“SWL-Child”) and a higher quality of overall life (“SWL-Life”). **See**Table S6 and S7 notes for model specification.

**Impact of Daughter's Education on Secondary Outcomes and Mediation**. We also evaluated the impact of educational attainment on parent-child interactions and support (Fig. 4; Table S6). Oldest daughters' college completion was significantly associated with a greater frequency of parent-child in-person interaction (β = 0.24, 95% CI: 0.15, 0.34) and a higher probability of parents reporting the receipt of gifts from adult children (β = 0.57, 95% CI: -0.17, 0.02), but did not significantly influence the likelihood of parents reporting financial support from their adult children (β = -0.07, 95% CI: -0.17, 0.02). We adjusted our primary models of parents' depressive symptoms to account for these factors, as they could serve as potential mediators. However, our estimates remained consistent even after further adjustment, suggesting a robust and direct influence of educational achievements on parental outcomes.

Finally, we evaluated the association between the daughter's college completion and parents' overall life satisfaction z-scores (Fig. 4; Table S7), which was generally weak and non-significant across all parents (β = -0.02, 95% CI: -0.22, 0.18 for parents with oldest daughters; β = 0.06, 95% CI: -0.17, 0.29) for parents with most highly educated daughters). While there was a negative association between college completion and satisfaction with child relations z-scores among fathers (β = -0.99, 95% CI: -1.31, -0.66 for oldest daughters; β = -0.91, 95% CI: -1.39, -0.42 for highest-educated daughters), the wide confidence intervals suggest considerable variability in this association.

**Sensitivity Tests**. We conducted several sensitivity analyses to assess the robustness of our results. First, we assessed alternative model specifications, including 1) randomly selecting an index child (Table S8) and 2) testing the proportion of children who completed college and the average years of schooling of all children aged 25+ within households (Table S9). Patterns found in our primary analyses were consistent across these alternative model specifications. Second, we evaluated the robustness of our instruments when varying birth cohort bounds in the construction of our instrument variable: (i.e. 5-year, 10-year, and 15-year birth cohort intervals) (Table S10). The partial F-statistics were relatively weaker when we used the 5-year and 15-year birth cohort bounds compared to the 10-year bounds (with the oldest child set as the index child), though the 5-year bounds yielded a better balance between the treated and control groups. Nonetheless, we consistently observed a decrease in parents' depressive symptoms across all specifications. Third, we assessed the impact of children's higher educational attainment on parental psychosocial well-being, distinguishing between 2-year/junior colleges and 4-year universities to understand the influence of different types of postsecondary education (Table S11). The 2-year/junior colleges are often considered lower-tier or alternatives to 4-year colleges in South Korea and, therefore, may not provide the same level of prestige or expected outcomes in terms of graduates' job prospects and the potential intergenerational spillover (Jung & Lee, 2016; Lee & Vignoles, 2022). Contrary to our initial expectations, we found a stronger association between parents' CES-D scores and the completion of 2-year colleges by the oldest children (β = -2.55, 95% CI: -3.82, -1.27) than with 4-year universities (β = -1.58, 95% CI: -2.60, -0.56), indicating fewer depressive symptoms, although the 95% confidence intervals overlapped. A stronger association was observed with the completion of 4-year universities by the highest-educated children, but the confidence intervals again significantly overlapped between 4-year and 2-year colleges.

## 4 Discussion

A growing body of research has increasingly turned its focus to how adult children's educational attainment and other dimensions of socio-economic status (SES) impact the health and well-being of their aging parents. Prior research has primarily relied on observational methods, which may overlook residual confounding factors. Our investigation contributes to this body of literature by using the data from the Korean Longitudinal Study of Aging and capitalizing on the 1993 higher education reform in South Korea as a natural experiment likely to have influenced the educational opportunities of participants' offspring. To our knowledge, this study is the first to assess the quasi-experimental impact of expanded higher education on the health outcomes of older adults, a departure from the traditionally studied effects of primary or secondary education expansion. Moreover, this research broadens the scope to consider settings like Korea, grappling with challenges in elderly care amidst a rapidly aging population and evolving gender norms. These shifts reflect changing caregiving dynamics that could influence the psychosocial well-being of parents.

Our findings indicate that college completion by a child due to the reform is associated with a large and significant reduction in depressive symptoms among parents. Increases in daughters' education levels had a notable impact in reducing CES-D scores for parents, with a stronger influence observed for mothers. Daughter's college completion correlates with a 6–25% decrease in the risk of elevated depressive symptoms for parents, with significant variation by the gender of the parent and the choice of the index child (e.g., oldest, highest educated). There was evidence of direct associations between daughter's college attainment and some measures of intergenerational contact and support (frequency of meeting and contact with, and receipt of gifts from, adult children), which could plausibly mediate the relationship between offspring education and parents' depressive symptoms. Associations with other secondary outcomes, including the receipt of financial transfers or reports of life satisfaction or satisfaction with adult children were null. Our primary results remained consistent even after further adjustment for secondary outcomes related to intergenerational contact and support.

Our findings also suggest heterogeneity in how daughter's college completion affects parents' psychosocial well-being, with significant differences based on parental characteristics such as urban versus rural residence and household assets. First-stage estimates suggested a stronger impact of policy reform on the children of rural parents – potentially because those in urban settings, often with higher SES – might have already supported college education for their children prior to the reform. The 1993 reform significantly improved college access in suburban and rural areas, given that major cities already had established and prestigious universities. Despite this, second-stage estimates suggested relatively greater returns to urban-dwelling parents. It could be that any impacts of offspring college attainment on rural parents' well-being are offset by the fact that most children with higher education in Korea tend to find employment in urban areas, which could reduce some more subtle aspects of intergenerational connection that were not captured in the data. The returns to daughter's college completion were also greater for those with lower (vs. higher) assets. According to the resource substitution theory (Ross & Mirowsky, 2011), education is especially valuable for those with fewer resources because it substitutes for financial capital, thus improving socioeconomic stability for those with lower SES. Therefore, for these parents, the educational advancements of their daughters may bring more substantial benefits by alleviating poverty and related stressors, which in turn could improve psychosocial well-being.

The variation in effects across different outcome domains is also worth noting. The heterogeneity in the effects of daughter's education on different dimensions of parental psychosocial well-being, such as depressive symptoms versus overall life satisfaction or satisfaction with children, may reflect how these outcomes respond to changes in children's status. Depressive symptoms are more likely to be shaped by recent relational dynamics and perceptions of children's current stability or success (Yan et al., 2025). In contrast, life satisfaction or satisfaction with children may represent more global, long-term evaluations influenced by cultural expectations, cumulative life events, and the full scope of parent-child relationships (Diener et al., 1985). As such, they may be less responsive to a single child's educational trajectory and more sensitive to broader family patterns.

Similarly, differences in the effects of adult children's education on intergenerational outcomes, such as the frequency of parent-child interactions or material support, may reflect prevailing social norms in South Korea. For example, the absence of increased financial transfers from daughters despite their higher educational attainment may be tied to cultural expectations that sons are primarily responsible for financial support in South Korea (Sung, 2018). In contrast, gifts – being more symbolic and emotionally expressive– may carry affective significance for parents and thus more directly influence their psychological well-being.

Although the primary analysis focused on daughters' education, our supplementary analysis incorporating all children (including sons) suggests that the relatively weak – or in some cases, adverse – associations between children's education and fathers' depressive symptoms are largely driven by the impact of sons' college attainment (Table S12). One explanation may be methodological: the 1993 education reform may have had a weaker first-stage effect for sons, as they were already more likely to attend college due to prevailing cultural preferences for investing in sons' education (Choi & Hwang, 2015), potentially reducing the marginal effect of the policy. Alternatively, the limited or negative impact of sons' education may reflect broader sociocultural dynamics in contemporary Korea. Historically, sons (particularly first-born sons) were expected to provide financial support, coreside with aging parents, and eventually inherit family assets (Sung, 2018), aligning with strong patrilineal norms. However, more recent evidence points to a loosening of these expectations: rates of coresidence and frequent contact with sons have declined, and intergenerational dynamics have become increasingly gender-neutral (Lim-Soh et al., 2025).

Furthermore, our findings suggest that sons' educational attainment, especially among oldest sons, may even increase the psychological burden on fathers. In the South Korean context, sons' college completion may evoke heightened financial and emotional strain among fathers due to enduring expectations around investment in sons' futures. For instance, prior research links private education costs to increased paternal depressive symptoms (Oh et al., 2020). In contrast, daughters, benefiting from rising educational attainment and labor force participation, may be better positioned to provide both emotional and instrumental support, which is closely tied to parents’ mental health and more often provided by daughters. These patterns reflect not only the persistence of gendered caregiving roles, but also the evolving mismatch between traditional filial expectations and the realities of intergenerational support in contemporary Korean society.

Our findings align with much of the existing research on the protective association between adult child education and parental depressive symptoms across various global context, albeit to varying degrees. Observational research from Europe (Sabater & Graham, 2016), Taiwan (Lee, 2018; Lee et al., 2017), and the United States (Yahirun et al., 2020), also demonstrate these protective associations, although direct comparisons are difficult to make because of the differences in exposure classification and study design. Three previous quasi-experimental studies (which focused on shifts at the earlier end of the education continuum) correspond with our findings to some extent: two identified protective effects of increased years of adult child schooling on depressive symptoms among older European (Torres et al., 2022) and older Mexican (Gutierrez et al., 2024) adults, while another study showed no association with depressive symptoms (but protective effects on life satisfaction) for older Chinese adults (Ma, 2019).

Differences between our results and those from prior studies could be due to several factors. First, in many European countries, state-sponsored social protections and a higher socioeconomic status might reduce the elderly's dependence on their children and vice-versa (Torres et al., 2022). Older adults in Korea (and similarly in China and Mexico) are often more economically vulnerable and dependent on financial support from their adult children (Kim and Cook, 2011; Kim et al., 2015). This vulnerability may lead to a greater beneficial impact of children's educational attainment on their well-being if educational attainment also yields improved opportunities for income and wealth generation (e.g., via higher-paying jobs).

Second, other quasi-experimental studies on this topic leveraged compulsory schooling laws that targeted lower or upper-secondary schooling. However, higher education reforms generally target older adolescents and adults, potentially providing more substantial and direct benefits in terms of career opportunities and earnings (Angrist et al., 1996; Autor, 2010). These outcomes of higher education reforms could have a more pronounced effect on parental well-being by enabling greater socioeconomic mobility and enhanced support capabilities of children, compared to the more foundational schooling driven by compulsory schooling laws. Finally, there are several cross-study differences in the measurement of depressive symptoms and adult child education, including the choice of the “index” child in quasi-experimental studies. We elaborate on the final point in SI Appendix 4.

Overall, our findings point to the large and multi-faceted impacts of daughter's college completion on parents' wellbeing. However, they also raise more opportunities to further investigate potential mechanisms that are rooted in the context of South Korea's evolving social and economic landscape. For instance, children who attain higher education often secure more stable and higher incomes, which can be a source of significant relief for their aging parents through both financial and psychosocial mechanisms (Ryff et al., 1994; Milkie et al., 2008). In a society where elders often rely on their offspring for financial security (Kim and Cook, 2011), well-educated children who are financially stable may provide better financial support and lessen the worry their parents might otherwise experience over monetary matters. Furthermore, in a culture that values filial piety such as South Korea, the educational success of children is seen as directly enhancing parental well-being and success (Fingerman et al., 2012; Park, 2018). This dynamic is particularly significant as many children, especially daughters who have benefited greatly from South Korea's higher education reform, are the first in their families to attend college. This success could not only boost parental pride but also foster a shared sense of achievement and emotional well-being within the family (Greenfield & Marks, 2006). Lastly, while traditional Korean norms have positioned sons as the primary caregivers, increased educational and career opportunities are enabling daughters to progressively assume significant roles in supporting their aging parents emotionally, psychologically, and with tangible gifts. This shift reflects not only changing societal norms regarding gender roles but also a broader transformation towards more egalitarian caregiving responsibilities within families.

## 5 Limitations

The findings of this study, which utilized a natural experiment through instrumental variable analysis, are specifically tied to the context of the 1993 higher education reform in South Korea, and thus have limited external validity. The results reflect the local average treatment effects for individuals born between 1964 and 1983 and may not be extendable to those born outside these years or in different geographic locations. Furthermore, the impact measured does not apply to “always-takers” – those who would have pursued higher education regardless of the reform and are often the most educated within their families – or “never-takers” – those who did not pursue college despite the reform. While various specifications of the index child (highest educated, oldest, random) were used to account for potential variability in our estimates, and consistent results were observed across these different specifications, there remains a possibility of residual variability in how children's education affects parental outcomes due to the presence of these specific groups. Furthermore, significant differences in parental characteristics pre-college expansion, such as education levels, urban or rural locality, and family size – likely influenced by broader socioeconomic shifts such as rapid industrialization – may have contributed to variations in college completion rates among their offspring, rather than educational policies alone. To account for these factors, we adjusted our models for observable covariates present before the educational expansion. We also tested different birth cohort bounds to effectively balance the distributions of these covariates between the treatment and control groups. Our decision to use a 10-year cohort bound struck a balance between achieving well-distributed covariates and retaining a large enough sample size to ensure the precision of our results. Lastly, the rapid expansion of higher education in South Korea raises concerns about potential disparities in the *quality* of education provided by newly established institutions compared to older, more established ones (Organisation for Economic Co-operation and Development Contributor, 2009). While some empirical data suggest that new institutions may not lag significantly behind established ones in key quality indicators (Ahn, 2011; Choi, 2015), such as student-faculty ratios, library resources per student, and overall investment per student, there may be other quality aspects that are harder to quantify for higher education. Although our study lacks detailed contextual data such as region, state, and school quality, which could influence the exposure of children to educational policies, we conducted stratification analysis by the type of higher education institutions offered in South Korea (4-year vs 2-year colleges), which are considered to provide differential levels of higher education quality in South Korea, and found no evidence of a differential impact on parental outcomes based on institutional quality. This suggests that while newer institutions may differ in certain quality indicators, these differences may not significantly affect the broader impacts of higher education on parental outcomes.

## 6 Conclusion

This study represents the first study, to our knowledge, that examines the impact of offspring college education on parents' late-life outcomes, utilizing the rapid educational expansion prompted by the 1993 higher education reform in Korea, in a quasi-experimental framework. Our findings contribute significant empirical evidence of an upward, intergenerational spillover effect of adult children's educational attainment on parental psychosocial well-being, particularly notable in daughters. These results are particularly relevant for policymakers in global contexts where compulsory education extends beyond secondary school and debates focus on the value of higher education. Amidst discussions on the cost of college attendance, our study underscores the link between children's educational attainment and parental health, highlighting how structures of social mobility can fundamentally influence the well-being of both generations. The opportunity for social advancement through education directly impacts parents, especially those from underprivileged backgrounds who are least likely to have college-educated children. This disadvantage can be exacerbated for parents when younger generations are unable to improve their socioeconomic status, deepening existing mental health disparities among older adults due to a cycle of restricted access to resources across generations. Therefore, policy interventions that aim to expand higher education access for lower socioeconomic status groups could have substantial benefits. Such policies could not only enhance social mobility but also improve population health, particularly impacting psychosocial well-being among parents in societies lacking robust elderly care systems. By improving educational opportunities, we can potentially mitigate some of the socioeconomic challenges faced by older adults and create a healthier, more equitable society for future generations.

## CRediT authorship contribution statement

**Ah-Reum Lee:** Writing – review & editing, Writing – original draft, Visualization, Validation, Software, Methodology, Investigation, Formal analysis, Data curation, Conceptualization. **K. Renata Flores Romero:** Writing – review & editing, Validation. **Jacqueline Torres:** Writing – review & editing, Writing – original draft, Supervision, Resources, Project administration, Funding acquisition, Conceptualization.

## Informed consent

Informed consent was not required for this study, as it involved the use of de-identified, publicly available secondary data.

## Conflict of interest statement

The authors whose names are listed above have no conflicts of interest to declare.

## Ethics approval

Ethical approval for this study was obtained from the Institutional Review Board at the University of California, San Francisco. This research involved secondary analysis of publicly available, de-identified data.

## Animal welfare

This research did not involve the use of animals.

## Funding

This work was supported by the 10.13039/100000049National Institute on Aging (R01AG072448). Jacqueline Torres reports additional funding from the 10.13039/100000049National Institute on Aging and the 10.13039/100000054National Cancer Institute outside of the reported research.

## Conflict of interest

The authors below declare no conflict of interest.

## Data Availability

Data will be made available on request.
