# Supplementary material for: Daughter's college completion and parents' psychosocial wellbeing: quasi-experimental evidence from South Korea
Source: SSM Popul Health. 2025 Jul 23;31:101846. doi: 10.1016/j.ssmph.2025.101846 (PMC12314392; doi:10.1016/j.ssmph.2025.101846)
Supplement: Multimedia component 1 [file mmc1.docx]

**Supporting Information for**

Daughter’s college completion and parents’ psychosocial wellbeing: quasi-experimental evidence from South Korea

**This file includes:**

Supporting information text

Figures S1 to S2

Tables S1 to S13

SI References

Supporting Information Text

**SI Appendix 1. Institutional Background for 1993 Higher Education Reform**

The 1993 higher education reform in South Korea marked a significant departure from the tightly regulated system that had previously kept college enrollment rates stable at around 35% since the late 1980s (Choi 1996). Under the previous regime, the government meticulously managed the capacity of college enrollment, allotting specific quotas across institutions. However, with the election of a new government in 1993, a wave of neoliberal reforms swept through the higher education sector (Lee and Sohn, 1994). The administration of President Kim Young-Sam introduced the 'Plan for the Promotion of College Enrollment Liberalization and the seminal '5·31 Education Reform Plan' in subsequent years (Lee 2000). These reforms were geared toward increasing 'autonomy and competition' and drastically changed the educational landscape. They ushered in a phase of deregulation that allowed for a surge in both the number of higher education institutions and student enrollment rates. New universities were encouraged to enter the market, provided they met minimum conditions, and this led to an unprecedented rise in the college enrollment rate by more than 25 percentage points from 1993 to 1997 (Choi and Lee 2017)**.**

The government’s initiative shifted the focus from equity in educational opportunity and demand fulfillment to one that emphasized the excellence and diversity of higher education, advocating for universities' autonomy and accountability. This period also saw the adoption of the 'University Establishment Standard Principle', leading to the rapid increase of specialized universities and a significant expansion in the number of colleges and universities (Choi and Lee 2017). The 'College Enrollment Liberalization policy continued under the subsequent Kim Dae-Jung government, which further liberalized enrollment by abolishing the 'College Student Enrollment Decree' and establishing the 'Higher Education Act and its enforcement decree.’ Despite some setbacks and unimplemented plans, the overarching aim was to prevent unrestrained expansion of enrollment while maintaining quality and efficiency through various reforms and structural adjustments. This reform era laid the groundwork for modernizing South Korean higher education, transforming it into a more open, diverse, and internationally competitive system that aligns more closely with global standards and practices. While these reforms received mixed reviews, they were undeniably pivotal in shaping the current state of higher education in South Korea.

**SI Appendix 2. Description of outcomes and covariates**

**Primary Outcomes**. Depressive symptoms among KLoSA respondents were measured with the 10-item Center for Epidemiologic Studies Depression Scale (CES-D 10) scores (Radloff, 1977), which comprise 10 items (range: 0-30 points) listed in the original 20-item version and has been validated (Irwin et al., 1999). The CES-D score in KLOSA is calculated as a composite score from the sum of ten items from the original 20-item version. The CES-D-10 was translated into Korean, and its reliability and validity have been confirmed (Cho and Kim, 1993; Kang et al., 2024).

The items measure depressive symptoms over the past two weeks, including: 1) Feeling depressed; 2) Trouble keeping mind on task; 3) Sleep was restless; 4) Felt happy (reverse coded); 5) Felt lonely; 6) Bothered by little things; 7) Everything was an effort; 8) Enjoyed life (reverse coded); 9) Felt fearful; 10) Could not get going, measured from Waves 1 through 4. Starting from Wave 5, the score is similarly calculated but includes three additional items (Felt sad; People were unfriendly; People disliked me) and excludes three prior items (Trouble keeping mind on task; Bothered by little things; Felt fearful). The response scale for each item is standardized to range from 0 (less negative feeling) to 3 (more negative feeling), adjusting from the original scale of 1 to 4. The total scores can range from 0 to 30, with higher scores indicating a greater frequency of negative feelings over the past two weeks.

In addition to the continuous CES-D scale, we constructed binary indicators to identify individuals at risk of depression, primarily using a cutoff score of 10 (Andresen et al., 1994; Björgvinsson et al., 2013) to aid interpretation and contextualize our findings. While a score of 10 or higher is often used to flag individuals who may require further evaluation, prior studies have also recommended alternative thresholds, such as 15 or 16, to improve specificity in identifying clinically significant symptoms (e.g., Björgvinsson et al., 2013). To assess the robustness of our findings, we therefore conducted sensitivity analyses using these higher cutoffs, which yielded substantively similar results.

**Secondary Outcomes**. We evaluated intergenerational support from the adult child to parents, including the frequency of contact and financial support. First, we used the frequency of interactions between an adult child and their parents and the exchange of financial and non-financial support to measure intergenerational support and relationships. The frequency of contact examines whether parents and, where applicable, their spouses engage with any of their children regularly, including various forms of communication such as in-person meetings, phone calls, or electronic messages. Financial support was assessed to determine if parents received any financial aid or non-financial gifts from their children or grandchildren within the past year. These dimensions of intergenerational support are critical indicators of the quality of life for older adults (Silverstein et al., 1997).

To quantify these relationships, respondents were asked to categorize the frequency of their contact with each child using a detailed scale ranging from daily interactions to never (1. almost every day or 4+ times a week/2. once a week/ 3. 2-3 times a week/ 4. once a month/5. twice a month/6. 1-2 times a year/7. 3-4 times a year/8. 5-6 times a year/9. rarely/10. never). We defined a binary variable, assigning a value of 1 to those parents who had contact with their children more than once a month, and a value of 0 to those with less frequent contact. Similarly, in-person meetings were categorized with a binary variable. For financial support, we assigned a value of 1 to respondents who had received regular or occasional financial help in the last year, and 0 if they did not. Non-financial gifts, such as clothing, leisure items, health products, household goods, and food, were also tracked with a binary variable to determine their presence or absence in the lives of the respondents. We adjust our models for these mediators of the association in our primary analysis.

For life satisfaction, respondents were asked to rate their overall satisfaction with life using the statement: “How satisfied are you with your overall quality of life (or how happy you feel)?” Additionally, they were asked a domain-specific question regarding their satisfaction with their relationships with their children, using the statement: “How satisfied are you with your relationship with your children?” Single-item measures of life satisfaction are widely used in panel studies and have demonstrated good psychometric properties and predictive validity (Cheung & Lucas, 2014). The answer scale for the original questions ranged from 0 (indicating dissatisfied) to 100 (indicating satisfied). Participants answered on a scale from 0 to 100 points, at intervals of 10 points, with higher scores indicating greater satisfaction. Although this variable has been reverse-coded to 0 (indicating satisfied) and 100 (indicating dissatisfied) in the harmonized KLoSA v2 (2006-2020), we utilize the original scale (0: completely dissatisfied – 100: completely satisfied) and standardized the variables into z-scores to improve the interpretability of the results.

**Covariates**. We controlled for a range of demographic, socioeconomic, and family-level characteristics of the respondents. Specifically, we accounted for respondents’ characteristics, including age (age in years at interview), gender (1. man/2. woman), marital status (1. married or living with a partner/2. separated, divorced, widowed, or never married), educational attainment (1. no education (illiterate)/2. no education (reading)/3. elementary school/4. middle school/5. high school/6. two-year college/7. college graduate/8. post-college (master's)/9. post-college (PhD)), and sample cohorts (1. Original KLoSA cohort/2. Refresher cohort); family-level characteristics, including the number of living children, children’s gender composition (measured as the percentage of sons), whether the respondent's father or mother is alive, household income (originally in 10,000 Korean Won, transformed into log), household assets (originally in 10,000 Korean Won, transformed into log), urban or rural residence (0. urban/1. rural), and regions (17 provinces and special cities); respondents’ health behaviors including currently smoking, currently drinking, and frequent exercise (+1/week); and early life characteristics such as parental education (1. less than lower secondary/2. upper secondary and vocational school/3. tertiary).

**SI Appendix 3. Assessment of Missing Data and Sensitivity Analysis Using Multiple Imputation**

To assess the robustness of our findings in light of missing data on key variables (approximately 5% of observations), we conducted two additional analyses. First, we tested whether the missing data were missing completely at random (MCAR) using Little’s MCAR test. The results indicated that the assumption of MCAR does not hold in our sample ($\chi^{2}=91.27, df=36, p<0.001)$. These results suggest that the missingness is not completely random, implying potential bias if only complete cases are analyzed. Given the rejection of MCAR, next, we conducted a sensitivity analysis using multiple imputation by chained equations (MICE) to account for missingness under the assumption that data are missing at random (MAR) (Azur et al., 2011). The imputation model included all variables used in the main analysis. We generated 10 imputed datasets and re-estimated the key models using the imputed data. The results from the imputed data were highly consistent with those from the complete-case analysis, suggesting that the observed relationships are not sensitive to the exclusion of cases with missing data. The results from the imputed analysis are presented in the Table below (next page), and support the robustness of our conclusions.

**Robustness of Effects of Daughter’s Education on Parental Depressive Symptoms: Complete-Case vs. Multiple Imputation Estimates**

|  |  |  |  |  |  |  |
| --- | --- | --- | --- | --- | --- | --- |
|  | **CES-D (0-30)** | | | | | |
|  | **No Imputation** | | | **Imputation** | | |
|  | CES-D (0-30) | | | CES-D (0-30) | | |
|  | Beta | [95% CI] | F | Beta | [95% CI] | F |
| Oldest daughter college completion | -1.376*** | (-2.423, -0.329) | 48.6 | -1.428*** | (-2.379, -0.477) | 48.6 |
| Oldest daughter years of schooling | -0.355** | (-0.627, -0.083) | 36.6 | -0.369*** | (-0.616, -0.122) | 36.6 |
|  | CES-D (0-30) | | | CES-D (0-30) | | |
|  | Beta | [95% CI] | F | Beta | [95% CI] | F |
| Highest-educated daughter college completion | -3.245*** | (-4.567, -1.923) | 49.0 | -3.161*** | (-4.467, -1.855) | 49.0 |
| Highest-educated daughter years of schooling | -0.877*** | (-1.243, -0.511) | 23.0 | -0.862*** | (-1.218, -0.507) | 23.0 |
|  | **At Risk of Depression (CES-D Score of 10 or above)** | | | | | |
|  | **No Imputation** | | | **Imputation** | | |
|  | Beta | [95% CI] | F | Beta | [95% CI] | F |
| Oldest daughter college completion | -0.048 | (-0.149, 0.054) | 48.6 | -0.057 | (-0.137, 0.024) | 48.6 |
| Oldest daughter years of schooling | -0.012 | (-0.039, 0.014) | 36.6 | -0.015 | (-0.036, 0.006) | 36.6 |
|  | **All parents** | | | **Fathers** | | |
|  | Beta | [95% CI] | F | Beta | [95% CI] | F |
| Highest-educated daughter college completion | -0.168*** | (-0.277, -0.058) | 49.0 | -0.173*** | (-0.286, -0.061) | 49.0 |
| Highest-educated daughter years of schooling | -0.045*** | (-0.076, -0.015) | 23.0 | -0.047*** | (-0.078, -0.017) | 23.0 |

Source/Notes: 1) Models include individuals aged 51 and above with at least one adult daughter who reported in the birth cohort affected by the reform (treatment group) or in the birth cohort from the preceding 10-year period (control group) from the Korean Longitudinal Study of Aging (KLOSA), 2006-2020 v2. 2) Models control for individual and family-level characteristics, including the respondent's age in years, gender, marital status, levels of education, urbanicity, household income, household assets, number of living children, children’s gender composition, whether the respondent's father or mother is alive, parental education, frequency of meeting or contact with children, whether receive or not financial and non-financial support from children, health behaviors including currently smoking and/or drinking, birth cohorts, and regional fixed effects. 3) Risk of depression was defined as having a score above the cut-off of 10 on the CES-D 10 scale. The total scores of the CES-D 10 scale range from 0 to 30, with higher scores indicating a greater frequency of negative feelings over the past two weeks. 4) Kleibergen-Paap Wald F-tests are used for F-statistics. Following conventional guidelines, a value above 10 is generally considered indicative of a sufficiently strong instrument (Stock & Yogo, 2005). *** p<0.01, ** p<0.05, * p<0.10.

**SI Appendix 4. Measurement of Offspring Education**

The categorization of offspring education in the literature varies, with each method chosen for its relevance to the research question and the hypothesized pathways through which children's education impacts parental outcomes. Therefore, the way we measure adult children’s education—whether as the oldest, the highest-educated, or by average schooling—may also account for the differences among studies. In our study, we found that using the highest-educated daughter as an index yielded less precise first-stage estimates but larger second-stage estimates. This pattern aligns with findings from Torres et al. (2022), where selecting the highest-educated child led to larger point estimates in associations with verbal fluency scores, potentially due to greater 'noncompliance' within this group. Since the highest-educated children may already surpass the educational requirements set by the reform and engage in additional educational activities independently of the reform, the impact of the education reform can be less pronounced for these groups. This situation can make the instrumental variable estimates more sensitive to even minor violations of the exclusion restriction (Angrist et al., 1993). Prior studies also reported smaller or null effects when using less educated children as the index child (Ma 2019; Ma et al., 2022), highlighting that the selection of the index child could substantially influence the robustness and magnitude of study estimates in education reform impacts. To mitigate potential biases from selecting specific children as the index, we also employed a randomized selection method. This approach revealed that randomly chosen indices resulted in generally smaller impacts on parental well-being, suggesting that previous effects may be partly due to characteristics specific to the oldest or most educated children.

For readers interested in further exploration, we present the previous literature on offspring education and the different methods of measuring offspring education below. This list provides an overview of significant studies and their approaches to understanding the impact of children's education on parental outcomes. It is important to note that this list is not exhaustive and serves as a starting point for deeper investigation into the topic. Furthermore, most studies incorporate multiple measures of offspring education in their analyses to account for different potential influences in their sensitivity analysis. For more details on individual research studies and additional references, please refer to the Reference list at the end of this document.

**1. Highest Educated Child:** Zimmer et al. (2002) and Zimmer et al. (2007) use the highest educational level of children living closest to the older adult, allowing for the investigation of the direct influence of the most proximal and potentially supportive child. Similarly, Yang et al. (2016) consider the educational attainment of the most highly educated co-resident child, which could reflect the maximum potential socioeconomic benefits accruing to the parent from their children. Sabater et al. (2020) also utilize the highest level of educational attainment among children, emphasizing the likely scenario where the most educated child contributes disproportionately to parental care or resources. Ma (2019) and Ma et al. (2022) use the highest-educated child as an index, assuming that the highest-educated children are the most important in shaping the health of older adults.

**2. Oldest Child:** Torssander (2013) analyzes the education level of the oldest child, which could be significant in cultures where the oldest child bears more responsibility for parental care. This approach is also used by Sabater et al. (2020) who look at the oldest child with the highest level of education, thus combining age and educational attainment to potentially identify the child most capable of supporting the parent. Torres et al. (2022) use the years of schooling attained by the oldest child as the primary exposure but also consider the highest-educated child as an alternative index. Similarly, Gutierrez et al. (2024) focus on the oldest child, examining how their educational attainment may set a precedent or influence the educational pathways of younger siblings, and tests the highest-educated child as an alternative.

**3. Proportional Measure:** Friedman & Mare (2014) and Lee C. (2018) employ proportional measures, where the education levels are categorized and analyzed as proportions of the total number of children. This method allows researchers to capture a more nuanced picture of how diverse educational achievements among children might collectively impact parental outcomes through a range of supportive behaviors or resources.

**4. Cumulative Measures:** Lee et al. (2017) use the average educational attainment of all living children, which provides a comprehensive view of the family's overall educational environment and its potential to affect parental well-being. This average could be reflective of the general socioeconomic status of the family, influencing the resources available for parental care. The approach by Yahirun et al. (2017) and Yahirun, Sheehan, & Mossakowski (2020) takes a cumulative measure of how many children have reached a specific educational threshold, exploring how widespread access to higher education within a family might impact parental health.

**SI Appendix 5. Key Assumptions for Instrumental Variable Analysis**

For the IV approach to be valid, several key assumptions must be satisfied:

*Relevance*

The instrument, exposure to the reform, must significantly influence children’s college completion (*Relevance*). This assumption is satisfied, as the partial F-statistic for the instrument exceeds the conventional threshold of 10 (Stock & Yogo, 2022). Furthermore, prior studies have confirmed that the 1993 higher education reform significantly increased access to higher education for cohorts born after 1974, as evidenced by analyses of Census data (Sohn and Lee, 2019). This provides additional support for the relevance of our chosen instrument.

*Exclusion Restriction*

The instrument should only affect parental mortality through its impact on children’s education, with no other causal pathways (*Exclusion Restriction*). While the exclusion restriction cannot be directly tested, several factors support its plausibility in the context of our study.

First, the 1993 higher education reform primarily increased college enrollment and completion, with no significant changes in the rates of high school graduation or graduate education, as shown in the supplementary table below using KLoSA data. This suggests that the instrument is unlikely to affect parental depressive symptoms through shifts in other levels of education, thereby reducing concerns about alternative educational pathways.

Association Between Exposure to the 1993 Higher Education Reform and Other Educational Outcomes

|  | **High School Completion** | **Graduate Education** |
| --- | --- | --- |
| Exposure to 1993 Higher Education Reform | 0.024 | 0.005 |
|  | (-0.015, 0.064) | (-0.014, 0.024) |
| F | 1.49 | 0.26 |

Source/Notes: 1) Models include respondents' adult children (aged 25 or above) from the Korean Longitudinal Study of Aging (KLOSA), 2006-2020 v2. 2) Models control for individual and family-level characteristics, including the respondent's age in years, gender, marital status, levels of education, urbanicity, household income, household assets, number of living children, children’s gender composition, whether the respondent's father or mother is alive, parental education, birth cohorts, and regional fixed effects. 3) Kleibergen-Paap Wald F-tests are used for F-statistics. Following conventional guidelines, a value above 10 is generally considered indicative of a sufficiently strong instrument (Stock & Yogo, 2005). *** p<0.01, ** p<0.05, * p<0.10.

Second, while financial burden from education spending could represent a potential mechanism linking the reform to parental well-being –– thus violating the exclusion restriction –– this concern appears to be minimal in the context of the 1990s. Private education expenditures and college tuition costs remained relatively stable during the mid-to-late 1990s and only began to increase sharply in the early 2000s, after the treated cohorts had already entered higher education (Kim et al., 2012). This temporal mismatch makes it unlikely that parents experienced significant financial stress as a direct consequence of the reform during the study period. If anything, any residual cost burdens would likely bias our estimates downward, reinforcing the interpretation that the observed associations represent a conservative estimate of the benefits of children’s college attainment.

Third, although the rapid expansion of higher education during the 1990s raises potential concerns about declines in institutional quality, which could present an alternative pathway for underestimating effects on parental health, prior research indicates that newly established institutions were generally comparable to older ones across key indicators such as student-faculty ratios, library resources, and investment per student (Choi, 2015; Ahn, 2011). Furthermore, our stratified analyses by institution type (4-year versus 2-year colleges) revealed no evidence of differential effects on parental depressive symptoms, implying that variation in college quality is unlikely to have systematically biased our estimates.

Fourth, while we acknowledge that labor market dynamics may shift the returns to education over time, direct evidence on declining wage returns to high school education in South Korea during the late 1990s and early 2000s remains limited. The 1997 Asian Financial Crisis introduced substantial macroeconomic disruptions; however, there is no clear indication that these shocks differentially impacted older or younger siblings based on their exposure to the reform or educational attainment in a way that would invalidate the exclusion restriction. Furthermore, because our outcome of interest is parental depressive symptoms, not labor market outcomes per se, potential spillovers from shifting wage returns would need to exert a substantial and systematic influence on parental mental health in order to pose a serious threat to identification.

Taken together, these findings provide strong support for the credibility of the exclusion restriction –– that is, the 1993 higher education reform appears to have influenced parental depressive symptoms primarily through its effect on children’s college completion. While we find no empirical evidence of alternative pathways, such as changes in other educational levels, educational quality, parental financial strain, or labor market competition, the possibility of unobserved channels cannot be entirely ruled out.

*Independence*

The independence assumption requires that the instrument be unrelated to unobserved confounders that jointly influence the treatment (college completion) and the outcome (parental depressive symptoms). While this assumption cannot be directly verified, its plausibility is supported by the fact that the 1993 higher education reform introduced a birth cohort discontinuity: it applied only to children born in 1974 or later. Although South Korea experienced broader socioeconomic changes during the 1990s, including economic liberalization and the 1997 financial crisis, these events were not specific to a single cohort and would have affected all families in a broadly similar way. Therefore, the assumption that there were no period shocks disproportionately influencing adjacent cohorts is more reasonable, making the independence assumption credible in our setting.

| a) Respondents with the oldest child  Exclude respondents missing covariates or outcome measures:  n=367  Exclude respondents whose child is neither in the treatment nor the control group: n=2,934  Exclude respondents whose child is less than 25 years old: n=299  Exclude respondents aged less than 50 or over 90: n=481  N=7,093  N=7,460  N=10,394  N=10,693  11,174 Respondents  (no proxy interviews),  KLOSA Wave 1-8 | b) Respondents with the highest-educated child  Exclude respondents missing covariates or outcome measures:  n=354  Exclude respondents whose child is neither in the treatment nor the control group: n=3,331  Exclude respondents whose child is less than 25 years old: n=301  Exclude respondents aged less than 50 or over 90: n=481  N=6,707  N=7,061  N=10,392  N=10,693  11,174 Respondents  (no proxy interviews),  KLOSA Wave 1-8 |
| --- | --- |

Fig. S1. Flowchart depicting analytic sample selection


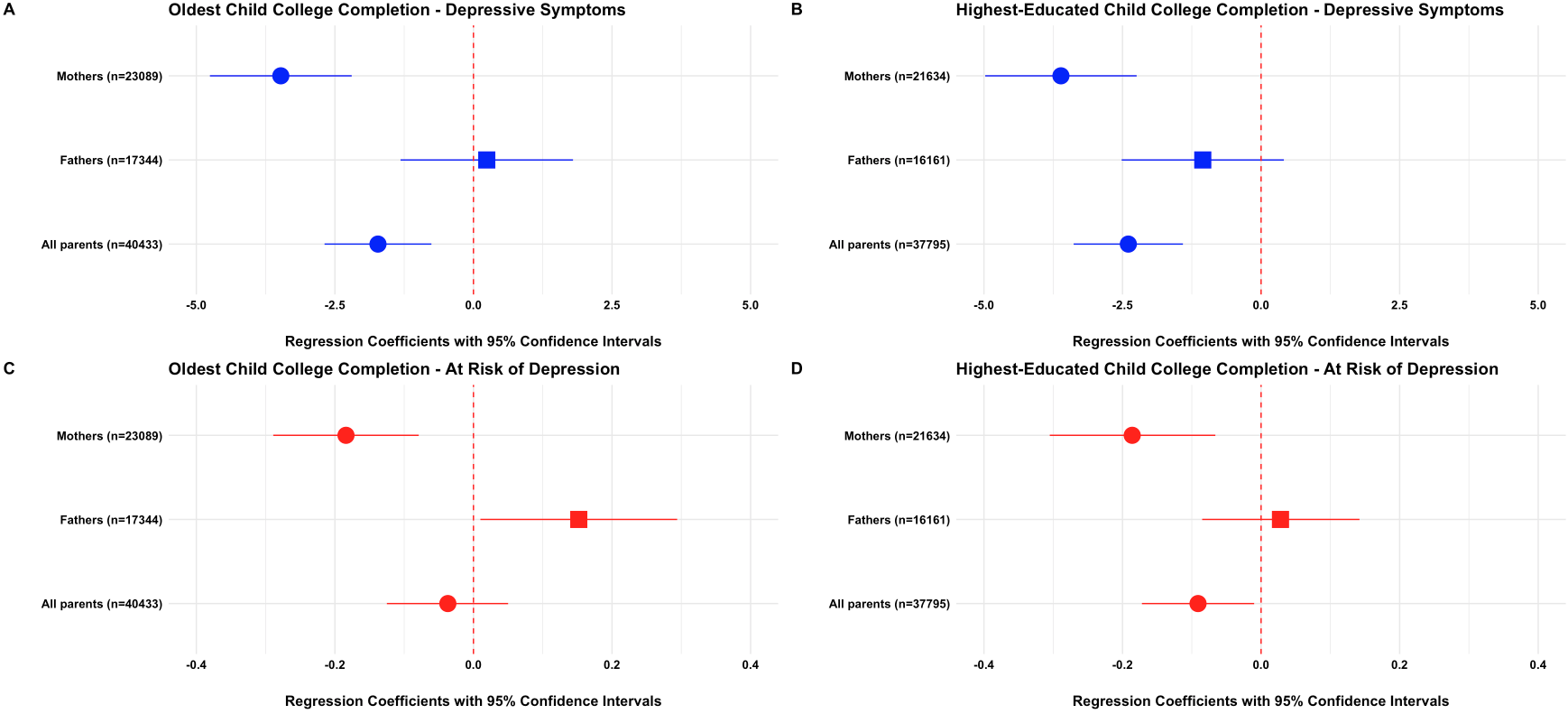


Fig. S2. Beta Coefficients and 95% Confidence Intervals from Two-Stage Least Squares Regressions Evaluating the Association between the College Completion of the Index Child (including both sons and daughters) and Older Parents’ Psychosocial Outcomes, by Type of Index Child

Notes: 1) Risk of depression was defined as having a score above the cut-off of 10 on the CES-D 10 scale. 2) The total scores of the CES-D 10 scale range from 0 to 30, with higher scores indicating a greater frequency of negative feelings over the past two weeks.

Table S1. Descriptive statistics of respondents aged 51+ years whose oldest child was in the first 10 birth cohorts to benefit from, or the preceding 10 birth cohorts to just miss benefitting from, the 1993 higher education reform, Korean Longitudinal Study on Aging (KLoSA), 2006-2020.

|  | **All** | **Treatment group** | **Control Group** |
| --- | --- | --- | --- |
|  |  | **(Child born** | **(Child born** |
|  |  | **1974-1983)** | **1964-1973)** |
| **Variable** | **Mean (SD)/%** | **Mean (SD)/%** | **Mean (SD)/%** |
| **Respondent characteristics** |  |  |  |
| Age (years) | 62.3 (8.2) | 54.8 (4.1) | 66.5 (6.8) |
| Female | 56% | 52% | 59% |
| High school or more | 32% | 54% | 22% |
| Married | 82% | 92% | 76% |
|  |  |  |  |
| **Household and family characteristics** |  |  |  |
| Rural | 25% | 18% | 29% |
| HH Income (Unit: 10 million KRW) | 1.3 (2.3) | 1.9 (2.5) | 1.0 (2.1) |
| HH Assets (Unit: 10 million KRW) | 9.2 (22.9) | 12.3 (27.9) | 7.5 (19.4) |
| Number of children | 3.2 (1.4) | 2.3 (0.8) | 3.7 (1.4) |
| Mother alive | 25% | 46% | 14% |
| Father alive | 4% | 14% | 3% |
|  |  |  |  |
| **Adult child educational attainment** |  |  |  |
| Child’s college completion rates | 61% | 80% | 47% |
| Child's years of schooling | 14.1 (2.6) | 15.1 (1.8) | 13.5 (2.7) |
|  |  |  |  |
| **Primary outcomes** |  |  |  |
| Depressive symptoms, CES-D 10 (range: 0-30)^2)^ | 6.6 (5.0) | 5.5 (4.3) | 7.1 (5.2) |
| At risk of depression (CES-D 10 scale over 10) | 21% | 14% | 25% |
|  |  |  |  |
| **Secondary outcomes** |  |  |  |
| Life satisfaction, overall (range: 0-100)^2)^ | 61.8 (20.3) | 65.2 (18.5) | 59.9 (21.0) |
| Satisfaction with child relations (range: 0-100)^2)^ | 72.7 (19.2) | 75.3 (17.6) | 71.5 (19.8) |
| Meeting in person (more than once a month) | 61% | 74% | 54% |
| Frequency of contact (more than once a month) | 93% | 97% | 91% |
| Financial transfer from child (last year) | 40% | 26% | 49% |
| Non-financial support (gifts) from child (last year) | 21% | 21% | 22% |
|  |  |  |  |
| **Observations (Person)** | **7,093** | **2,525** | **4,568** |
| **Observations (Person-Times)** | **39,465** | **16,795** | **22,670** |

Notes: 1) The CES-D 10 scale measures depressive symptoms over the past two week, with scores ranging from 0 (indicating no symptoms) to 30. 2) Life satisfaction (overall, with child relations) ranged from 0 (completely dissatisfied) to 100 (completely satisfied), so that higher scores indicate a higher quality of life.

Table S2. Beta Coefficients and 95% Confidence Intervals from First-Stage Regression Predicting Educational Attainment of the Index Child Based on Exposure to the 1993 Reform, Overall and by Child’s Sex/Gender

|  | **College completion** | | | | | | | | |
| --- | --- | --- | --- | --- | --- | --- | --- | --- | --- |
|  | **Oldest children (n=7,093)** | | | **Oldest sons (n=3,736)** | | | **Oldest daughters (n=3,355)** | | |
|  | College completion | | | College completion | | | College completion | | |
|  | Beta | [95% CI] | F | Beta | [95% CI] | F | Beta | [95% CI] | F |
| Exposure to 1993 Higher Education Reform | 0.146*** | (0.107, 0.185) | 53.4 | 0.106*** | (0.054, 0.158) | 15.8 | 0.194*** | (0.136, 0.253) | 42.4 |
|  |  |  |  |  |  |  |  |  |  |
|  | **Highest-educated children (n=6,707)** | | | **Highest-educated sons (n=3,968)** | | | **Highest-educated daughters (n=2,737)** | | |
|  | College completion | | | College completion | | | College completion | | |
|  | Beta | [95% CI] | F | Beta | [95% CI] | F | Beta | [95% CI] | F |
| Exposure to 1993 Higher Education Reform | 0.131*** | (0.098, 0.164) | 61.4 | 0.114*** | (0.072, 0.155) | 29.2 | 0.164*** | (0.110 0.218) | 36.0 |
|  |  |  |  |  |  |  |  |  |  |
|  | **Years of Schooling** | | | | | | | | |
|  | **Oldest children (n=7,093)** | | | **Oldest sons (n=3,736)** | | | **Oldest daughters (n=3,355)** | | |
|  | Years of schooling | | | Years of schooling | | | Years of schooling | | |
|  | Beta | [95% CI] | F | Beta | [95% CI] | F | Beta | [95% CI] | F |
| Exposure to 1993 Higher Education Reform | 0.544*** | (0.357, 0.731) | 32.5 | 0.326** | (0.064, 0.587) | 6.0 | 0.791*** | (0.526, 1.056) | 34.3 |
|  |  |  |  |  |  |  |  |  |  |
|  | **Highest-educated children (n=6,707)** | | | **Highest-educated sons (n=3,968)** | | | **Highest-educated daughters (n=2,737)** | | |
|  | Years of schooling | | | Years of schooling | | | Years of schooling | | |
|  | Beta | [95% CI] | F | Beta | [95% CI] | F | Beta | [95% CI] | F |
| Exposure to 1993 Higher Education Reform | 0.533*** | (0.355, 0.711) | 34.4 | 0.464*** | (0.237, 0.691) | 20.3 | 0.656*** | (0.370 0.941) | 20.3 |
|  |  |  |  |  |  |  |  |  |  |

Source/Notes: 1) Models include respondents' adult children (aged 25 or above) who reported in the birth cohort affected by the reform (treatment group) or in the birth cohort from the preceding 10-year period (control group) from the Korean Longitudinal Study of Aging (KLOSA), 2006-2020 v2. 2) Models control for individual and family-level characteristics, including the respondent's age in years, gender, marital status, levels of education, urbanicity, household income, household assets, number of living children, children’s gender composition, whether the respondent's father or mother is alive, parental education, birth cohorts, and regional fixed effects. 3) Kleibergen-Paap Wald F-tests are used for F-statistics. Following conventional guidelines, a value above 10 is generally considered indicative of a sufficiently strong instrument & Yogo, 2005). *** p<0.01, ** p<0.05, * p<0.10.

Table S3. Beta Coefficients and 95% Confidence Intervals from Two-Stage Least Squares (2SLS) Regression for Predicting Parents’ Depressive Symptoms and Risk of Depression Based on Daughters’ Education, Overall

|  |  | | |  | **All parents (n=19,009)** | | |  |  |  |
| --- | --- | --- | --- | --- | --- | --- | --- | --- | --- | --- |
|  | CES-D (0-30) | | |  | At risk of depression  (CESD $\geq10$) | | | At elevated risk of depression  (CESD $\geq15$) | | |
|  | Beta | [95% CI] | F |  | Beta | [95% CI] | F |  |  |  |
| Oldest daughter college completion | -1.505*** | (-2.507, -0.504) | 48.6 |  | -0.111*** | (-0.194, -0.029) | 48.6 |  | 0.003 | (-0.045, 0.052) |
| Oldest daughter years of schooling | -0.738*** | (-0.984, -0.491) | 36.6 |  | -0.047*** | (-0.067, -0.027) | 36.6 |  | 0.001 | (-0.012, 0.013) |
|  |  | | |  | **All parents (n=15,192)** | | |  |  |  |
|  | CES-D (0-30) | | |  | At risk of depression  (CESD $\geq10$) | | | At elevated risk of depression  (CESD $\geq15$) | | |
|  | Beta | [95% CI] | F |  | Beta | [95% CI] | F |  |  |  |
| Highest-educated daughter college completion | -3.245*** | (-4.567, -1.923) | 49.0 |  | -0.168*** | (-0.277, -0.058) | 49.0 |  | -0.072** | (-0.137, -0.008) |
| Highest-educated daughter years of schooling | -0.877*** | (-1.243, -0.511) | 23.0 |  | -0.045*** | (-0.076, -0.015) | 23.0 |  | -0.020** | (-0.037, -0.002) |

Source/Notes: 1) Models include individuals aged 51 and above with at least one adult daughter who reported in the birth cohort affected by the reform (treatment group) or in the birth cohort from the preceding 10-year period (control group) from the Korean Longitudinal Study of Aging (KLOSA), 2006-2020 v2. 2) Models control for individual and family-level characteristics, including the respondent's age in years, gender, marital status, levels of education, urbanicity, household income, household assets, number of living children, children’s gender composition, whether the respondent's father or mother is alive, parental education, frequency of meeting or contact with children, whether receive or not financial and non-financial support from children, health behaviors including currently smoking and/or drinking, birth cohorts, and regional fixed effects. 3) Risk of depression was defined as having a CES-D 10 score above the cutoff of 10, and elevated risk as having a score above 15. The total scores of the CES-D 10 scale range from 0 to 30, with higher scores indicating a greater frequency of negative feelings over the past two weeks. 4) Kleibergen-Paap Wald F-tests are used for F-statistics. Following conventional guidelines, a value above 10 is generally considered indicative of a sufficiently strong instrument (Stock & Yogo, 2005). *** p<0.01, ** p<0.05, * p<0.10.

Table S4. Beta Coefficients and 95% Confidence Intervals from 2SLS Regression for Predicting Parents’ Depressive Symptoms and Risk of Depression Based on Daughters’ Education, Overall and by Parents’ Sex/Gender

|  | |  | | | | | | **CES-D Scores (0-30)** | | | | |  | | | |
| --- | --- | --- | --- | --- | --- | --- | --- | --- | --- | --- | --- | --- | --- | --- | --- | --- |
|  | | **All parents (n=19,009)** | | | | | | **Fathers (n=8,183)** | | | | | **Mothers (n=10,826)** | | | |
|  | | CES-D (0-30) | | | | | | CES-D (0-30) | | | | | CES-D (0-30) | | | |
|  | | Beta | | [95% CI] | F | | | Beta | | [95% CI] | F | | Beta | [95% CI] | F | |
| Oldest daughter college completion | | -1.505*** | | (-2.507, -0.504) | 48.6 | | | -0.424 | | (-1.548, 0.701) | 36.5 | | -2.402*** | (-3.950 -0.853) | 42.1 | |
| Oldest daughter years of schooling | | -0.738*** | | (-0.984, -0.491) | 36.6 | | | -0.367** | | (-0.671, -0.062) | 25.2 | | -0.972*** | (-0.671, -0.062) | 32.4 | |
|  | | **All parents (n=15,192)** | | | | | | **Fathers (n=6,570)** | | | | | **Mothers (n=8,622)** | | | |
|  | | CES-D (0-30) | | | | | | CES-D (0-30) | | | | | CES-D (0-30) | | | |
|  | | Beta | | [95% CI] | F | | | Beta | | [95% CI] | F | | Beta | [95% CI] | F | |
| Highest-educated daughter college completion | | -3.245*** | | (-4.567, -1.923) | 49.0 | | | -1.998* | | (-4.117, 0.122) | 33.1 | | -4.341*** | (-6.059, -2.623) | 45.3 | |
| Highest-educated daughter years of schooling | | -0.877*** | | (-1.243, -0.511) | 23.0 | | | -0.510* | | (-1.065, 0.045) | 16.6 | | -1.216*** | (-1.720 -0.712) | 20.9 | |
|  | **At Risk of Depression (CES-D Score of 10 or above)** | | | | | | | | | | | | | | | |
|  | | **All parents (n=19,009)** | | | | | | **Fathers (n=8,183)** | | | | | **Mothers (n=10,826)** | | | |
|  | At risk of depression | | | | | | At risk of depression | | | | | | At risk of depression | | | |
|  | Beta | | [95% CI] | | | F | Beta | | [95% CI] | | | F | Beta | [95% CI] | | F |
| Oldest daughter college completion | -0.111*** | | (-0.194, -0.029) | | | 48.6 | -0.008 | | (-0.108, 0.092) | | | 36.5 | -0.188*** | (-0.298, -0.078) | | 42.1 |
| Oldest daughter years of schooling | -0.047*** | | (-0.067, -0.027) | | | 36.6 | -0.016 | | (-0.041, 0.010) | | | 25.2 | -0.066*** | (-0.092, -0.040) | | 32.4 |
|  | **All parents (n=15,192)** | | | | | | **Fathers (n=6,570)** | | | | | | **Mothers (n=8,622)** | | | |
|  | At risk of depression | | | | | | At risk of depression | | | | | | At risk of depression | | | |
|  | Beta | | [95% CI] | | | F | Beta | | [95% CI] | | | F | Beta | [95% CI] | | F |
| Highest-educated daughter college completion | -0.168*** | | (-0.277, -0.058) | | | 49.0 | -0.057 | | (-0.225, 0.111) | | | 33.1 | -0.250*** | (-0.387, -0.114) | | 45.3 |
| Highest-educated daughter years of schooling | -0.045*** | | (-0.076, -0.015) | | | 23.0 | -0.015 | | (-0.058, 0.029) | | | 16.6 | -0.070*** | (-0.110 -0.031) | | 20.9 |
|  | **At Elevated Risk of Depression (CES-D Score of 15 or above)** | | | | | | | | | | | | | | | |
|  | **All parents (n=19,009)** | | | | | | **Fathers (n=8,183)** | | | | | | **Mothers (n=10,826)** | | | |
|  | At risk of depression | | | | | | At risk of depression | | | | | | At risk of depression | | | |
|  | Beta | | [95% CI] | | | F | Beta | | [95% CI] | | | F | Beta | [95% CI] | | F |
| Oldest daughter college completion | 0.003 | | (-0.045, 0.052) | | | 48.6 | 0.034 | | (-0.045, 0.113) | | | 36.5 | -0.061** | (-0.121, -0.001) | | 42.1 |
| Oldest daughter years of schooling | 0.001 | | (-0.012, 0.013) | | | 36.6 | 0.009 | | (-0.012, 0.030) | | | 25.2 | -0.031*** | (-0.047, -0.015) | | 32.4 |
|  | **All parents (n=15,192)** | | | | | | **Fathers (n=6,570)** | | | | | | **Mothers (n=8,622)** | | | |
|  | At risk of depression | | | | | | At risk of depression | | | | | | At risk of depression | | | |
|  | Beta | | [95% CI] | | | F | Beta | | [95% CI] | | | F | Beta | [95% CI] | | F |
| Highest-educated daughter college completion | -0.072** | | (-0.137, -0.008) | | | 49.0 | -0.056 | | (-0.159, 0.046) | | | 33.1 | -0.100** | (-0.183, -0.017) | | 45.3 |
| Highest-educated daughter years of schooling | -0.020** | | (-0.037, -0.002) | | | 23.0 | -0.014 | | (-0.041, 0.012) | | | 16.6 | -0.028** | (-0.051, -0.005) | | 20.9 |

Source/Notes: 1) Models include individuals aged 51 and above with at least one adult daughter who reported in the birth cohort affected by the reform (treatment group) or in the birth cohort from the preceding 10-year period (control group) from the Korean Longitudinal Study of Aging (KLOSA), 2006-2020 v2. 2) Models control for individual and family-level characteristics, including the respondent's age in years, gender, marital status, levels of education, urbanicity, household income, household assets, number of living children, children’s gender composition, whether the respondent's father or mother is alive, parental education, frequency of meeting or contact with children, whether receive or not financial and non-financial support from children, health behaviors including currently smoking and/or drinking, birth cohorts, and regional fixed effects. 3) Risk of depression was defined as having a CES-D 10 score above the cutoff of 10, and elevated risk as having a score above 15. The total scores of the CES-D 10 scale range from 0 to 30, with higher scores indicating a greater frequency of negative feelings over the past two weeks. 4) Kleibergen-Paap Wald F-tests are used for F-statistics. Following conventional guidelines, a value above 10 is generally considered indicative of a sufficiently strong instrument (Stock & Yogo, 2005). *** p<0.01, ** p<0.05, * p<0.10.

Table S5. Beta Coefficients and 95% Confidence Intervals from 2SLS Regression for Predicting Parents’ Depressive Symptoms Based on Daughters’ Education, by Parents’ Socioeconomic Characteristics

|  | **Oldest daughters** | | | | | |
| --- | --- | --- | --- | --- | --- | --- |
|  | **Rural (n=5,110)** | | | **Urban (n=13,899)** | | |
|  | CES-D (0-30) | | | CES-D (0-30) | | |
|  | Beta | [95% CI] | F | Beta | [95% CI] | F |
| Oldest daughter college completion | -1.066* | (-2.180 0.048) | 31.2 | -1.322* | (-2.695, 0.050) | 27.4 |
| Oldest daughter years of schooling | -0.283* | (-0.583, 0.016) | 24.0 | -0.346* | (-0.711, 0.020) | 13.1 |
|  | **Below Median Household Assets (n=9,401)** | | | **Above Median Household Assets (n=9,608)** | | |
|  | CES-D (0-30) | | | CES-D (0-30) | | |
|  | Beta | [95% CI] | F | Beta | [95% CI] | F |
| Oldest daughter college completion | -2.938*** | (-4.391, -1.484) | 42.8 | 0.814 | (-0.698, 2.327) | 31.1 |
| Oldest daughter years of schooling | -0.745*** | (-1.120 -0.371) | 34.4 | 0.219 | (-0.202, 0.640) | 21.2 |
|  | **Less than High School (n=12,561)** | | | **High School or Above (n=6,448)** | | |
|  | CES-D (0-30) | | | CES-D (0-30) | | |
|  | Beta | [95% CI] | F | Beta | [95% CI] | F |
| Oldest daughter college completion | -2.041*** | (-3.145, -0.937) | 42.6 | 0.759 | (-2.088, 3.606) | 12.7 |
| Oldest daughter years of schooling | -0.545*** | (-0.839, -0.252) | 32.1 | 0.167 | (-0.461, 0.794) | 10.2 |
|  | **Highest-educated daughters** | | | | | |
|  | **Rural (n=3,854)** | | | **Urban (n=11,338)** | | |
|  | CES-D (0-30) | | | CES-D (0-30) | | |
|  | Beta | [95% CI] | F | Beta | [95% CI] | F |
| Highest-educated daughter college completion | -2.078*** | (-3.027, -1.130) | 72.1 | -3.717*** | (-6.417, -1.017) | 13.1 |
| Highest-educated daughter years of schooling | -0.580*** | (-0.853, -0.306) | 24.0 | -0.966*** | (-1.686, -0.246) | 6.0 |
|  | **Below Median Household Assets (n=7,426)** | | | **Above Median Household Assets (n=7,766)** | | |
|  | CES-D (0-30) | | | CES-D (0-30) | | |
|  | Beta | [95% CI] | F | Beta | [95% CI] | F |
| Highest-educated daughter college completion | -3.724*** | (-5.453, -1.994) | 40.1 | -1.798* | (-3.896, 0.299) | 36.9 |
| Highest-educated daughter years of schooling | -1.073*** | (-1.616, -0.530) | 19.2 | -0.462* | (-0.997, 0.073) | 16.9 |
|  | **Less than High School (n=9,352)** | | | **High School or Above (n=5,840)** | | |
|  | CES-D (0-30) | | | CES-D (0-30) | | |
|  | Beta | [95% CI] | F | Beta | [95% CI] | F |
| Highest-educated daughter college completion | -2.814*** | (-3.912, -1.716) | 59.6 | -5.945 | (-16.959, 5.070) | 3.5 |
| Highest-educated daughter years of schooling | -0.797*** | (-1.116, -0.477) | 28.4 | -1.123 | (-2.603, 0.357) | 2.0 |

Source/Notes: 1) Models include individuals aged 51 and above with at least one adult daughter who reported in the birth cohort affected by the reform (treatment group) or in the birth cohort from the preceding 10-year period (control group) from the Korean Longitudinal Study of Aging (KLOSA), 2006-2020 v2. 2) Models control for individual and family-level characteristics, including the respondent's age in years, gender, marital status, levels of education, urbanicity, household income, household assets, number of living children, children’s gender composition, whether the respondent's father or mother is alive, parental education, frequency of meeting or contact with children, whether receive or not financial and non-financial support from children, health behaviors including currently smoking and/or drinking, birth cohorts, and regional fixed effects. 3) Kleibergen-Paap Wald F-tests are used for F-statistics. Following conventional guidelines, a value above 10 is generally considered indicative of a sufficiently strong instrument (Stock & Yogo, 2005). *** p<0.01, ** p<0.05, * p<0.10.

Table S6. Beta Coefficients and 95% Confidence Intervals from 2SLS Regression for Predicting Intergenerational Relationships and Supports from Children Based on Daughters’ College Completion, Overall and by Parents’ Socioeconomic Characteristics

|  | **Oldest daughters** | | | | | | | | |
| --- | --- | --- | --- | --- | --- | --- | --- | --- | --- |
|  | **All parents** | | | **Fathers** | | | **Mothers** | | |
|  | Beta | [95% CI] | F | Beta | [95% CI] | F | Beta | [95% CI] | F |
| **Meet often** | 0.243*** | (0.149, 0.337) | 48.6 | 0.199*** | (0.061, 0.336) | 36.5 | 0.266*** | (0.122, 0.410) | 42.1 |
| Observations | 19085 | | | 8205 | | | 10880 | | |
| **Contact often** | 0.056** | (0.000 0.112) |  | 0.041 | (-0.035, 0.117) |  | 0.071** | (0.006, 0.135) |  |
| Observations | 19085 | | | 8205 | | | 10880 | | |
| **Financial support** | -0.074 | (-0.167, 0.018) |  | -0.096 | (-0.233, 0.042) |  | 0.071** | (-0.152, 0.121) |  |
| Observations | 19085 | | | 8205 | | | 10880 | | |
| **Gift** | 0.572*** | (-0.167, 0.018) |  | 0.640*** | (0.472, 0.808) |  | 0.536*** | (0.378, 0.694) |  |
| Observations | 16945 | | | 7210 | | | 9735 | | |
|  | **Highest-educated daughter** | | | | | | | | |
|  | **All parents** | | | **Fathers** | | | **Mothers** | | |
|  | Beta | [95% CI] | F | Beta | [95% CI] | F | Beta | [95% CI] | F |
| **Meet often** | 0.136** | (0.001, 0.272) | 49.0 | 0.163 | (-0.039, 0.365) | 33.1 | 0.103 | (-0.077, 0.282) | 45.27 |
| Observations | 15247 | | | 6589 | | | 8658 | | |
| **Contact often** | 0.053* | (-0.006, 0.111) |  | 0.025 | (-0.061, 0.112) |  | 0.070 | (-0.017, 0.158) |  |
| Observations | 15247 | | | 6589 | | | 8658 | | |
| **Financial support** | -0.020 | (-0.148, 0.108) |  | 0.033 | (-0.164, 0.230) |  | -0.012 | (-0.155, 0.131) |  |
| Observations | 15247 | | | 6589 | | | 8658 | | |
| **Gift** | 0.630*** | (0.498, 0.762) |  | 0.793*** | (0.558, 1.028) |  | 0.505*** | (0.338, 0.671) |  |
| Observations | 13236 | | | 5650 | | | 7586 | | |

Source/Notes: 1) Models include individuals aged 51 and above with at least one adult daughter who reported in the birth cohort affected by the reform (treatment group) or in the birth cohort from the preceding 10-year period (control group) from the Korean Longitudinal Study of Aging (KLOSA), 2006-2020 v2. 2) Models control for individual and family-level characteristics, including the respondent's age in years, gender, marital status, levels of education, urbanicity, household income, household assets, number of living children, children’s gender composition, whether the respondent's father or mother is alive, parental education, health behaviors including currently smoking and/or drinking, birth cohorts, and regional fixed effects. *** p<0.01, ** p<0.05, * p<0.10.

Table S7. Beta Coefficients and 95% Confidence Intervals from 2SLS Regression for Predicting Parents’ Life Satisfaction Based on Daughters’ Education, Overall and by Parents’ Sex/Gender

|  | **Satisfaction with Overall Quality of Life Z-Scores** | | | | | | | | |
| --- | --- | --- | --- | --- | --- | --- | --- | --- | --- |
|  | **All parents (n=19,076)** | | | **Fathers (n=8,200)** | | | **Mothers (n=10,876)** | | |
|  | Overall life satisfaction | | | Overall life satisfaction | | | Overall life satisfaction | | |
|  | Beta | [95% CI] | F | Beta | [95% CI] | F | Beta | [95% CI] | F |
| Oldest daughter college completion | -0.020 | (-0.217, 0.177) | 45.0 | -0.262 | (-0.580 0.055) | 34.2 | 0.108 | (-0.167, 0.383) | 38.9 |
| Oldest daughter years of schooling | -0.005 | (-0.056, 0.046) | 32.9 | -0.069 | (-0.154, 0.017) | 23.0 | 0.027 | (-0.043, 0.098) | 28.8 |
|  | **All parents (n=15,244)** | | | **Fathers (n=6,588)** | | | **Mothers (n=8,656)** | | |
|  | Overall life satisfaction | | | Overall life satisfaction | | | Overall life satisfaction | | |
|  | Beta | [95% CI] | F | Beta | [95% CI] | F | Beta | [95% CI] | F |
| Highest-educated daughter college completion | 0.062 | (-0.169, 0.293) | 39.6 | -0.133 | (-0.508, 0.242) | 25.8 | 0.155 | (-0.152, 0.462) | 39.5 |
| Highest-educated daughter years of schooling | 0.017 | (-0.046, 0.080) | 17.7 | -0.034 | (-0.132, 0.064) | 11.2 | 0.043 | (-0.042, 0.129) | 18.7 |
|  | **Satisfaction with Children Relations Z-Scores** | | | | | | | | |
|  | **All parents (n=13,190)** | | | **Fathers (n=4,937)** | | | **Mothers (n=8,253)** | | |
|  | Satisfied with child relations | | | Satisfied with child relations | | | Satisfied with child relations | | |
|  | Beta | [95% CI] | F | Beta | [95% CI] | F | Beta | [95% CI] | F |
| Oldest daughter college completion | -0.207* | (-0.445, 0.031) | 45.0 | -0.988*** | (-1.316, -0.659) | 34.2 | 0.289* | (-0.053, 0.630) | 38.9 |
| Oldest daughter years of schooling | -0.053* | (-0.115, 0.008) | 32.9 | -0.258*** | (-0.351, -0.165) | 23.0 | 0.073* | (-0.014, 0.161) | 28.8 |
|  | **All parents (n=10,490)** | | | **Fathers (n=3,877)** | | | **Mothers (n=6,613)** | | |
|  | Satisfied with child relations | | | Satisfied with child relations | | | Satisfied with child relations | | |
|  | Beta | [95% CI] | F | Beta | [95% CI] | F | Beta | [95% CI] | F |
| Highest-educated daughter college completion | -0.112 | (-0.391, 0.167) | 39.6 | -0.906*** | (-1.389, -0.422) | 25.8 | 0.334* | (-0.011, 0.678) | 39.5 |
| Highest-educated daughter years of schooling | -0.030 | (-0.105, 0.045) | 17.7 | -0.231*** | (-0.360 -0.103) | 11.2 | 0.094* | (-0.004, 0.191) | 18.7 |

Source/Notes: 1) Models include individuals aged 51 and above with at least one adult daughter who reported in the birth cohort affected by the reform (treatment group) or in the birth cohort from the preceding 10-year period (control group) from the Korean Longitudinal Study of Aging (KLOSA), 2006-2020 v2. 2) Models control for individual and family-level characteristics, including the respondent's age in years, gender, marital status, levels of education, urbanicity, household income, household assets, number of living children, children’s gender composition, whether the respondent's father or mother is alive, parental education, frequency of meeting or contact with children, whether receive or not financial and non-financial support from children, health behaviors including currently smoking and/or drinking, birth cohorts, and regional fixed effects. 3) Life satisfaction was recoded as standardized z-scores, with higher scores indicating a higher quality of life. 4) Kleibergen-Paap Wald F-tests are used for F-statistics. Following conventional guidelines, a value above 10 is generally considered indicative of a sufficiently strong instrument (Stock & Yogo, 2005). *** p<0.01, ** p<0.05, * p<0.10.

Table S8. Beta Coefficients and 95% Confidence Intervals from 2SLS Regression for Predicting Parents’ Depressive Symptoms Based on Randomly Selected Daughters’ Education, Overall and by Parents’ Sex/Gender

|  | **CES-D (0-30)** | | | | | |
| --- | --- | --- | --- | --- | --- | --- |
|  | **All parents** | | **Fathers** | | **Mothers** | |
|  | CES-D (0-30) | | CES-D (0-30) | | CES-D (0-30) | |
|  | Beta | [95% CI] | Beta | [95% CI] | Beta | [95% CI] |
| Oldest daughter college completion | -1.620*** | (-2.787, -0.498) | -0.477 | (-2.063, 1.112) | -2.609*** | (-4.132, -1.186) |

**Source/Notes**: 1) Models include individuals aged 51 and above with at least one adult daughter who reported in the birth cohort affected by the reform (treatment group) or in the birth cohort from the preceding 10-year period (control group) from the Korean Longitudinal Study of Aging (KLOSA), 2006-2020 v2. 2) Models control for individual and family-level characteristics, including the respondent's age in years, gender, marital status, levels of education, urbanicity, household income, household assets, number of living children, children’s gender composition, whether the respondent's father or mother is alive, parental education, frequency of meeting or contact with children, whether receive or not financial and non-financial support from children, health behaviors including currently smoking and/or drinking, birth cohorts, and regional fixed effects. 3) Kleibergen-Paap Wald F-tests are used for F-statistics. Following conventional guidelines, a value above 10 is generally considered indicative of a sufficiently strong instrument (Stock & Yogo, 2005). *** p<0.01, ** p<0.05, * p<0.10.

Table S9. Beta Coefficients and 95% Confidence Intervals from 2SLS Regression for Predicting Parents’ Depressive Symptoms Based on a Cumulative Measure of Offspring Education (Proportion of Children who Completed College, Average Years of Schooling), Overall and by Parents’ Sex/Gender

|  | **All parents (n=52,509)** | | | **Fathers (n=52,509)** | | | **Mothers (n=52,509)** | | |
| --- | --- | --- | --- | --- | --- | --- | --- | --- | --- |
|  | CES-D (0-30) | | | CES-D (0-30) | | | CES-D (0-30) | | |
|  | Beta | [95% CI] | F | Beta | [95% CI] | F | Beta | [95% CI] | F |
| Proportion of children who completed college | -3.078*** | (-4.171, -1.986) | 67.2 | -0.130 | (-1.742, 1.482) | 56.1 | -6.227*** | (-7.894, -4.561) | 50.3 |
| Average years of schooling | -0.911*** | (-1.238, -0.583) | 33.5 | -0.037 | (-0.497, 0.423) | 31.2 | -1.949*** | (-2.540 -1.358) | 21.3 |

**Source/Notes**: 1) Models include individuals aged 51 and above with at least one adult child who reported in the birth cohort affected by the reform (treatment group) or in the birth cohort from the preceding 10-year period (control group) from the Korean Longitudinal Study of Aging (KLOSA), 2006-2020 v2. 2) Models control for individual and family-level characteristics, including the respondent's age in years, gender, marital status, levels of education, urbanicity, household income, household assets, number of living children, children’s gender composition, whether the respondent's father or mother is alive, parental education, frequency of meeting or contact with children, whether receive or not financial and non-financial support from children, health behaviors including currently smoking and/or drinking, birth cohorts, and regional fixed effects. 3) We instrumented the proportion of children who completed college with the proportion of children exposed to the 1993 higher education reform, and we used the average years of required schooling as an instrument for the average years of schooling of all children aged 25 and older within households. 4) Kleibergen-Paap Wald F-tests are used for F-statistics. Following conventional guidelines, a value above 10 is generally considered indicative of a sufficiently strong instrument (Stock & Yogo, 2005). *** p<0.01, ** p<0.05, * p<0.10.

Table S10. Comparison of Different Ranges of Birth Cohort Bounds for the Instrument: 5-Year vs. 10-Year vs. 15-Year Birth Cohort Bounds in the 2SLS Regression for Predicting Parents’ Depressive Symptoms Based on Index Child’s Education

|  | **College completion** | | | | | | | | |
| --- | --- | --- | --- | --- | --- | --- | --- | --- | --- |
| **First-stage results** | **5-Year (n=28,513)** | | | **10-Year (n=37,934)** | | | **15-Year (n=47,048)** | | |
|  | Beta | [95% CI] | F | Beta | [95% CI] | F | Beta | [95% CI] | F |
| Exposure to 1993, Higher Education Reform | 0.086*** | (0.044, 0.128) | 16.4 | 0.140*** | (0.107, 0.185) | 85.0 | 0.138*** | (0.101, 0.174) | 54.3 |
|  |  |  |  |  |  |  |  |  |  |
| **Second-stage results** | **5-Year (n=28,406)** | | | **10-Year (n=37,795)** | | | **15-Year (n=46,871)** | | |
|  | CES-D (0-30) | | | CES-D (0-30) | | | CES-D (0-30) | | |
|  | Beta | [95% CI] |  | Beta | [95% CI] |  | Beta | [95% CI] |  |
| Oldest child college completion | -2.381** | (-4.318, -0.444) |  | -1.723*** | (-2.687, -0.759) |  | -2.369*** | (-3.352, -1.387) |  |
|  |  |  |  |  |  |  |  |  |  |
| **First-stage results** | **5-Year (n=28,513)** | | | **10-Year (n=38,580)** | | | **15-Year (n=47,048)** | | |
|  | Beta | [95% CI] | F | Beta | [95% CI] | F | Beta | [95% CI] | F |
| Exposure to 1993 Higher Education Reform | 0.135*** | (0.099, 0.170) | 54.8 | 0.139*** | (0.098, 0.164) | 73.1 | 0.137*** | (0.109, 0.166) | 89.8 |
|  |  |  |  |  |  |  |  |  |  |
| **Second-stage results** | **5-Year (n=28,406)** | | | **10-Year (n=37,795)** | | | **15-Year (n=42,592)** | | |
|  | CES-D (0-30) | | | CES-D (0-30) | | | CES-D (0-30) | | |
|  | Beta | [95% CI] |  | Beta | [95% CI] |  | Beta | [95% CI] |  |
| Highest-educated child college completion | -2.143*** | (-3.254, -1.032) |  | -2.395*** | (-3.381, -1.410) |  | -3.178*** | (-4.187, -2.169) |  |
|  | **Years of schooling** | | | | | | | | |
| **First-stage results** | **5-Year (n=28,513)** | | | **10-Year (n=37,934)** | | | **15-Year (n=47,048)** | | |
|  | Beta | [95% CI] | F | Beta | [95% CI] | F | Beta | [95% CI] | F |
| Exposure to 1993 Higher Education Reform | 0.302*** | (0.111, 0.493) | 9.6 | 0.480*** | (0.098, 0.164) | 35.2 | 0.488*** | (0.304, 0.672) | 18.5 |
|  |  |  |  |  |  |  |  |  |  |
| **Second-stage results** | **5-Year (n=28,406)** | | | **10-Year (n=37,795)** | | | **15-Year (n=46,871)** | | |
|  | CES-D (0-30) | | | CES-D (0-30) | | | CES-D (0-30) | | |
|  | Beta | [95% CI] |  | Beta | [95% CI] |  | Beta | [95% CI] |  |
| Oldest child years of schooling | -0.678** | (-1.232, -0.123) |  | -0.504*** | (-0.791, -0.217) |  | -0.592*** | (-0.901, -0.283) |  |
| **First-stage results** | **5-Year (n=27,813)** | | | **10-Year (n=38,580)** | | | **15-Year (n=42,746)** | | |
|  | Beta | [95% CI] | F | Beta | [95% CI] | F | Beta | [95% CI] | F |
| Exposure to 1993 Higher Education Reform | 0.488*** | (0.304, 0.672) | 27 | 0.494*** | (0.355, 0.711) | 25.5 | 0.478*** | 0.478 | 35.2 |
|  |  |  |  |  |  |  |  |  |  |
| **Second-stage results** | **5-Year (n=28,513)** | | | **10-Year (n=38,448)** | | | **15-Year (n=42,592)** | | |
|  | CES-D (0-30) | | | CES-D (0-30) | | | CES-D (0-30) | | |
|  | Beta | [95% CI] |  | Beta | [95% CI] |  | Beta | [95% CI] |  |
| Highest-educated child years of schooling | -0.592*** | (-0.901, -0.283) |  | -0.671*** | (-0.953, -0.389) |  | -0.914*** | (-1.216, -0.612) |  |

**Source/Notes**: 1) Models include individuals aged 51 and above with at least one adult child who reported in the birth cohort affected by the reform (treatment group) or in the birth cohort from the preceding 10-year period (control group) from the Korean Longitudinal Study of Aging (KLOSA), 2006-2020 v2. 2) Models control for individual and family-level characteristics, including the respondent's age in years, gender, marital status, levels of education, urbanicity, household income, household assets, number of living children, children’s gender composition, whether the respondent's father or mother is alive, parental education, frequency of meeting or contact with children, whether receive or not financial and non-financial support from children, health behaviors including currently smoking and/or drinking, birth cohorts, and regional fixed effects. 3) Kleibergen-Paap Wald F-tests are used for F-statistics. Following conventional guidelines, a value above 10 is generally considered indicative of a sufficiently strong instrument (Stock & Yogo, 2005). *** p<0.01, ** p<0.05, * p<0.10.

Table S11. Beta Coefficients and 95% Confidence Intervals from 2SLS Regression for Predicting Parents’ Depressive Symptoms Based on Daughters’ Education, Overall and by Types of Colleges

|  | **Oldest Child** | | | | | | | | |
| --- | --- | --- | --- | --- | --- | --- | --- | --- | --- |
|  | **All parents (n=36,906)** | | | **Fathers (n=15,730)** | | | **Mothers (n=21,176)** | | |
|  | CES-D (0-30) | | | CES-D (0-30) | | | CES-D (0-30) | | |
|  | Beta | [95% CI] | F | Beta | [95% CI] | F | Beta | [95% CI] | F |
| Oldest child 4-year college completion | -1.580*** | (-2.598, -0.561) | 44.7 | 0.591 | (-1.017, 2.199) | 31.7 | -3.477*** | (-4.897, -2.057) | 39.3 |
|  | **All parents (n=34,331)** | | | **Fathers (n=14,636)** | | | **Mothers (n=19,695)** | | |
|  | CES-D (0-30) | | | CES-D (0-30) | | | CES-D (0-30) | | |
|  | Beta | [95% CI] | F | Beta | [95% CI] | F | Beta | [95% CI] | F |
| Oldest child 2-year college completion | -2.549*** | (-3.824, -1.274) | 32.7 | -0.140 | (-1.905, 1.624) | 24.2 | -4.862*** | (-6.744, -2.980) | 28.4 |
|  | **Highest-educated child** | | | | | | | | |
|  | **All parents (n=19,327)** | | | **Fathers (n=7,724)** | | | **Mothers (n=11,603)** | | |
|  | CES-D (0-30) | | | CES-D (0-30) | | | CES-D (0-30) | | |
|  | Beta | [95% CI] | F | Beta | [95% CI] | F | Beta | [95% CI] | F |
| Highest-educated child 4-year college completion | -2.567*** | (-3.639, -1.495) | 68.3 | -1.070 | (-2.634, 0.494) | 53.6 | -4.010*** | (-5.554, -2.466) | 55.6 |
|  | **All parents (n=12,411)** | | | **Fathers (n=4,727)** | | | **Mothers (n=7,684)** | | |
|  | CES-D (0-30) | | | CES-D (0-30) | | | CES-D (0-30) | | |
|  | Beta | [95% CI] | F | Beta | [95% CI] | F | Beta | [95% CI] | F |
| Highest-educated child 2-year college completion | -1.894*** | (-2.909, -0.878) | 69.5 | -1.206* | (-2.608, 0.197) | 49.1 | -2.422*** | (-3.657, -1.186) | 66.5 |

**Source/Notes**: 1) Models include individuals aged 51 and above with at least one adult child who reported in the birth cohort affected by the reform (treatment group) or in the birth cohort from the preceding 10-year period (control group) from the Korean Longitudinal Study of Aging (KLOSA), 2006-2020 v2. 2) Models control for individual and family-level characteristics, including the respondent's age in years, gender, marital status, levels of education, urbanicity, household income, household assets, number of living children, children’s gender composition, whether the respondent's father or mother is alive, parental education, frequency of meeting or contact with children, whether receive or not financial and non-financial support from children, health behaviors including currently smoking and/or drinking, birth cohorts, and regional fixed effects. 3) Kleibergen-Paap Wald F-tests are used for F-statistics. Following conventional guidelines, a value above 10 is generally considered indicative of a sufficiently strong instrument (Stock & Yogo, 2005). *** p<0.01, ** p<0.05, * p<0.10.

Table S12. Beta Coefficients and 95% Confidence Intervals from 2SLS Regression for Predicting Parents’ Depressive Symptoms and Risk of Depression Based on Child’s Education, Overall and by Parents’ Sex/Gender

|  | |  | | | | | | **CES-D Scores (0-30)** | | | | |  | | | |
| --- | --- | --- | --- | --- | --- | --- | --- | --- | --- | --- | --- | --- | --- | --- | --- | --- |
|  | | **All parents (n=37,795)** | | | | | | **Fathers (n=16,161)** | | | | | **Mothers (n=21,634)** | | | |
|  | | CES-D (0-30) | | | | | | CES-D (0-30) | | | | | CES-D (0-30) | | | |
|  | | Beta | | [95% CI] | F | | | Beta | | [95% CI] | F | | Beta | [95% CI] | F | |
| Oldest child college completion | | -1.723*** | | (-2.687, -0.759) | 53.4 | | | 0.240 | | (-1.316, 1.796) | 24.8 | | -3.476*** | (-4.755, -2.197) | 59.0 | |
| Oldest child years of schooling | | -0.504*** | | (-0.791, -0.217) | 32.5 | | | 0.076 | | (-0.417, 0.568) | 13.5 | | -0.971*** | (-1.360 -0.583) | 35.6 | |
|  | | **All parents (n=38,448)** | | | | | | **Fathers (n=16,497)** | | | | | **Mothers (n=21,951)** | | | |
|  | | CES-D (0-30) | | | | | | CES-D (0-30) | | | | | CES-D (0-30) | | | |
|  | | Beta | | [95% CI] | F | | | Beta | | [95% CI] | F | | Beta | [95% CI] | F | |
| Highest-educated child college completion | | -2.395*** | | (-3.381, -1.410) | 61.4 | | | -1.052 | | (-2.515, 0.411) | 43.0 | | -3.611*** | (-4.977, -2.245) | 51.9 | |
| Highest-educated child years of schooling | | -0.671*** | | (-0.953, -0.389) | 34.4 | | | -0.281 | | (-0.671, 0.109) | 29.6 | | -1.058*** | (-1.490 -0.627) | 24.2 | |
|  | **At Risk of Depression (CES-D Score of 10 or above)** | | | | | | | | | | | | | | | |
|  | **All parents (n=37,795)** | | | | | | **Fathers (n=16,161)** | | | | | | **Mothers (n=21,634)** | | | |
|  | At risk of depression | | | | | | At risk of depression | | | | | | At risk of depression | | | |
|  | Beta | | [95% CI] | | | F | Beta | | [95% CI] | | | F | Beta | [95% CI] | | F |
| Oldest child college completion | -0.037 | | (-0.125, 0.050) | | | 53.4 | 0.152** | | (0.010 0.294) | | | 24.8 | -0.184*** | (-0.289, -0.079) | | 59.0 |
| Oldest child years of schooling | -0.011 | | (-0.036, 0.015) | | | 32.5 | 0.048** | | (0.002, 0.094) | | | 13.5 | -0.051*** | (-0.081, -0.021) | | 35.6 |
|  | **All parents (n=38,591)** | | | | | | **Fathers (n=16,542)** | | | | | | **Mothers (n=22,049)** | | | |
|  | At risk of depression | | | | | | At risk of depression | | | | | | At risk of depression | | | |
|  | Beta | | [95% CI] | | | F | Beta | | [95% CI] | | | F | Beta | [95% CI] | | F |
| Highest-educated child college completion | -0.091** | | (-0.172, -0.010) | | | 61.4 | 0.028 | | (-0.085, 0.142) | | | 43.0 | -0.186*** | -0.186 | | 51.9 |
| Highest-educated child years of schooling | -0.026** | | (-0.048, -0.003) | | | 34.4 | 0.008 | | (-0.023, 0.038) | | | 29.6 | -0.054*** | -0.054 | | 24.2 |

Source/Notes: 1) Models include individuals aged 51 and above with at least one adult child who reported in the birth cohort affected by the reform (treatment group) or in the birth cohort from the preceding 10-year period (control group) from the Korean Longitudinal Study of Aging (KLOSA), 2006-2020 v2. 2) Models control for individual and family-level characteristics, including the respondent's age in years, gender, marital status, levels of education, urbanicity, household income, household assets, number of living children, children’s gender composition, whether the respondent's father or mother is alive, parental education, frequency of meeting or contact with children, whether receive or not financial and non-financial support from children, health behaviors including currently smoking and/or drinking, birth cohorts, and regional fixed effects. 3) Risk of depression was defined as having a score above the cut-off of 10 on the CES-D 10 scale. The total scores of the CES-D 10 scale range from 0 to 30, with higher scores indicating a greater frequency of negative feelings over the past two weeks. 4) Kleibergen-Paap Wald F-tests are used for F-statistics. Following conventional guidelines, a value above 10 is generally considered indicative of a sufficiently strong instrument (Stock & Yogo, 2005). *** p<0.01, ** p<0.05, * p<0.10.

Table S13. Beta Coefficients and 95% Confidence Intervals from 2SLS Regression for Predicting Parents’ Life Satisfaction Based on Child’s Education, Overall and by Parents’ Sex/Gender

|  | **Satisfaction with Overall Quality of Life Z-Scores** | | | | | | | | |
| --- | --- | --- | --- | --- | --- | --- | --- | --- | --- |
|  | **All parents (n=37,921)** | | | **Fathers (n=16,197)** | | | **Mothers (n=21,724)** | | |
|  | Overall life satisfaction | | | Overall life satisfaction | | | Overall life satisfaction | | |
|  | Beta | [95% CI] | F | Beta | [95% CI] | F | Beta | [95% CI] | F |
| Oldest child college completion | -0.019 | (-0.219, 0.182) | 48.0 | -0.339** | (-0.664, -0.013) | 32.9 | 0.129 | (-0.110 0.368) | 43.3 |
| Oldest child years of schooling | -0.005 | (-0.064, 0.053) | 23.2 | -0.107* | (-0.216, 0.002) | 13.6 | 0.036 | (-0.031, 0.103) | 22.2 |
|  | **All parents (n=38,576)** | | | **Fathers (n=16,535)** | | | **Mothers (n=22,041)** | | |
|  | Overall life satisfaction | | | Overall life satisfaction | | | Overall life satisfaction | | |
|  | Beta | [95% CI] | F | Beta | [95% CI] | F | Beta | [95% CI] | F |
| Highest-educated child college completion | -0.097 | (-0.255, 0.062) | 69.9 | -0.186 | (-0.446, 0.074) | 53.2 | -0.093 | (-0.278, 0.092) | 60.3 |
| Highest-educated child years of schooling | -0.027 | (-0.072, 0.018) | 21.3 | -0.050 | (-0.121, 0.021) | 17.6 | -0.027 | (-0.082, 0.028) | 17.7 |
|  | **Satisfaction with Children Relations Z-Scores** | | | | | | | | |
|  | **All parents (n=26,345)** | | | **Fathers (n=9,611)** | | | **Mothers (n=16,734)** | | |
|  | Satisfied with child relations | | | Satisfied with child relations | | | Satisfied with child relations | | |
|  | Beta | [95% CI] | F | Beta | [95% CI] | F | Beta | [95% CI] | F |
| Oldest child college completion | -0.117 | (-0.343, 0.108) | 48.0 | -0.733*** | (-1.149, -0.316) | 32.9 | 0.237 | (-0.074, 0.548) | 43.3 |
| Oldest child years of schooling | -0.034 | (-0.100 0.031) | 23.2 | -0.232*** | (-0.369, -0.095) | 13.6 | 0.066 | (-0.020 0.152) | 22.2 |
|  | **All parents (n=26,798)** | | | **Fathers (n=9,824)** | | | **Mothers (n=16,974)** | | |
|  | Satisfied with child relations | | | Satisfied with child relations | | | Satisfied with child relations | | |
|  | Beta | [95% CI] | F | Beta | [95% CI] | F | Beta | [95% CI] | F |
| Highest-educated child college completion | -0.226** | (-0.439, -0.013) | 69.9 | -0.536*** | (-0.910 -0.163) | 53.2 | -0.042 | (-0.299, 0.216) | 60.3 |
| Highest-educated child years of schooling | -0.064** | (-0.124, -0.003) | 21.3 | -0.144*** | (-0.250 -0.039) | 17.6 | -0.012 | (-0.090 0.065) | 17.7 |

Source/Notes: 1) Models include individuals aged 51 and above with at least one adult child who reported in the birth cohort affected by the reform (treatment group) or in the birth cohort from the preceding 10-year period (control group) from the Korean Longitudinal Study of Aging (KLOSA), 2006-2020 v2. 2) Models control for individual and family-level characteristics, including the respondent's age in years, gender, marital status, levels of education, urbanicity, household income, household assets, number of living children, children’s gender composition, whether the respondent's father or mother is alive, parental education, frequency of meeting or contact with children, whether receive or not financial and non-financial support from children, health behaviors including currently smoking and/or drinking, birth cohorts, and regional fixed effects. 3) Life satisfaction was recoded as standardized z-scores, with higher scores indicating a higher quality of life. 4) Kleibergen-Paap Wald F-tests are used for F-statistics. Following conventional guidelines, a value above 10 is generally considered indicative of a sufficiently strong instrument (Stock & Yogo, 2005). *** p<0.01, ** p<0.05, * p<0.10.

**SI References**

1. Choi, K.-S. (1996). The impact of shifts in supply of college graduates: Repercussion of Educational reform in Korea. Economics of Education Review, 15(1), 1–9. <https://doi.org/10.1016/0272-7757(95)00031-3>
2. Lee, C.-S., & Sohn, H.-S. (1994). South Korea in 1993: The Year of the Great Reform. Asian Survey, 34(1), 1–9. <https://doi.org/10.1525/as.1994.34.1.00p0343w>
3. Lee, J.-K. (2000). Main reform on higher education systems in Korea [La reforma principal de los sistemas de educación superior en Corea]. Korean Educational Development Institute, Division of Educational Policy Research. Received September 20, 2000; accepted for publication October 11, 2000.
4. Choi, K. S., & Lee, B. K. (2017). Trends and Issues of the Higher Education Reform Policy in Korea: With Focus on the Regulation on Enrollment Size (대학정원정책을 중심으로 본 한국의 대학구조개혁정책의 변화와 쟁점). Korean Journal of General Education, 11(1), 313-363. Available in Korean only.
5. Radloff, L. S. (1977). The CES-D Scale: A Self-Report Depression Scale for Research in the General Population. Applied Psychological Measurement, 1(3), 385–401. <https://doi.org/10.1177/014662167700100306>
6. CHO, M. J., & KIM, K. H. (1998). Use of the Center for Epidemiologic Studies Depression (CES-D) Scale in Korea. The Journal of Nervous & Mental Disease, 186(5), 304–310. <https://doi.org/10.1097/00005053-199805000-00007>
7. Kang, S. J., Hwang, J., Kim, D., & Kim, B. (2024). Regional differences in the effects of healthy aging on depressive symptoms: A Korean longitudinal study of aging (2006–2020). Frontiers in Public Health, 12, 1256368. <https://doi.org/10.3389/fpubh.2024.1256368>
8. Andresen, E. M., Malmgren, J. A., Carter, W. B., & Patrick, D. L. (1994). Screening for Depression in Well Older Adults: Evaluation of a Short Form of the CES-D. American Journal of Preventive Medicine, 10(2), 77–84. <https://doi.org/10.1016/S0749-3797(18)30622-6>
9. Björgvinsson, T., Kertz, S. J., Bigda-Peyton, J. S., McCoy, K. L., & Aderka, I. M. (2013). Psychometric Properties of the CES-D-10 in a Psychiatric Sample. Assessment, 20(4), 429–436. https://doi.org/10.1177/1073191113481998
10. Silverstein M, Bengtson VL. Intergenerational Solidarity and the Structure of Adult Child‐Parent Relationships in American Families. American Journal of Sociology. 1997;103(2):429-460. doi: <https://doi.org/10.1086/231213>
11. Cheung, F., & Lucas, R. E. (2014). Assessing the validity of single-item life satisfaction measures: Results from three large samples. Quality of Life Research, 23(10), 2809–2818. <https://doi.org/10.1007/s11136-014-0726-4>
12. Azur MJ, Stuart EA, Frangakis C, Leaf PJ. (2011). Multiple imputation by chained equations: what is it and how does it work? International Journal of Methods in Psychiatric Research, 20(1), 40–49. <https://doi.org/10.1002/mpr.329>
13. Torres, J. M., Yang, Y., Rudolph, K. E., Meza, E., Glymour, M. M., & Courtin, E. (2022). Adult Child Schooling and Older Parents’ Cognitive Outcomes in the Survey of Health, Aging and Retirement in Europe (SHARE): A Quasi-Experimental Study. American Journal of Epidemiology, 191(11), 1906–1916. <https://doi.org/10.1093/aje/kwac151>
14. Silverstein, M., & Bengtson, V. L. (1997). Intergenerational Solidarity and the Structure of Adult Child‐Parent Relationships in American Families. American Journal of Sociology, 103(2), 429–460. <https://doi.org/10.1086/231213>
15. Angrist, J., Imbens, G., & Rubin, D. (1996). Identification of Causal Effects Using Instrumental Variables. Journal of the American Statistical Association, 91(434), 444--455. <https://doi.org/10.2307/2291629>
16. Ma, M. (2019). Does children’s education matter for parents’ health and cognition? Evidence from China. Journal of Health Economics, 66, 222–240. <https://doi.org/10.1016/j.jhealeco.2019.06.004>
17. Ma, Y., Ma, Z., & Yang, M. (2022). Does Adult Children’s Education Increase Parents’ Longevity in China? International Journal of Environmental Research and Public Health, 19(23), 15530-. <https://doi.org/10.3390/ijerph192315530>
18. Zimmer, Z., Hermalin, A. I., & Lin, H. S. (2002). Whose education counts? The added impact of adult-child education on physical functioning of older Taiwanese. Journals of Gerontology Series B: Psychological Sciences and Social Sciences, 57(1), S23–S32. <https://doi.org/10.1093/geronb/57.1.S23>
19. Z. Zimmer, L.G. Martin, M.B. Ofstedal, Y.L. Chuang, Education of adult children and mortality of their elderly parents in Taiwan. Demography 44, 289–305 (2007). <https://doi.org/10.1353/dem.2007.0020>
20. Yang, L., Martikainen, P., & Silventoinen, K. (2016). Effects of Individual, Spousal, and Offspring Socioeconomic Status on Mortality Among Elderly People in China. Journal of epidemiology, 26(11), 602–609. <https://doi.org/10.2188/jea.JE20150252>
21. Sabater, A., Graham, E., & Marshall, A. (2020). Does having highly educated adult children reduce mortality risks for parents with low educational attainment in Europe? Ageing and Society, 40(12), 2635–2670. <https://doi.org/10.1017/S0144686X19000795>
22. Torssander, J. (2013). From child to parent? The significance of children's education for their parents' longevity. Demography, 50(2), 637–659. <https://doi.org/10.1007/s13524-012-0155-3>
23. Gutierrez, S., Courtin, E., Glymour, M. M., & Torres, J. M. (2024). Does schooling attained by adult children affect parents’ psychosocial well-being in later life? Using Mexico’s 1993 compulsory schooling law as a quasi-experiment. SSM - Population Health, 25, 101616–101616. <https://doi.org/10.1016/j.ssmph.2024.101616>
24. Friedman, E.M., Mare, R.D. (2014). The schooling of offspring and the survival of parents. Demography 51(4), 1271–1293. <https://doi.org/10.1007/s13524-014-0303-z>.
25. Lee C. (2018). Adult children’s educational attainment and the cognitive trajectories of older parents in South Korea. Social Science & Medicine (1982), 209, 76–85. <https://doi.org/10.1016/j.socscimed.2018.05.026>
26. Lee, C., Glei, D. A., Goldman, N., & Weinstein, M. (2017). Children’s Education and Parents’ Trajectories of Depressive Symptoms. Journal of Health and Social Behavior, 58(1), 86–101. <https://doi.org/10.1177/0022146517690200>
27. Yahirun, J. J., Sheehan, C. M., & Hayward, M. D. (2017). Adult children’s education and changes to parents’ physical health in Mexico. Social Science & Medicine (1982), 181, 93–101. <https://doi.org/10.1016/j.socscimed.2017.03.034>
28. Yahirun, J. J., Vasireddy, S., & Hayward, M. D. (2020). The Education of Multiple Family Members and the Life-Course Pathways to Cognitive Impairment. The Journals of Gerontology. Series B, Psychological Sciences and Social Sciences, 75(7), e113–e128. <https://doi.org/10.1093/geronb/gbaa039>
29. Stock, J. H., & Yogo, M. (2005). Testing for weak instruments in linear IV regression. In D. W. K. Andrews & J. H. Stock (Eds.), Identification and Inference for Econometric Models: Essays in Honor of Thomas Rothenberg (pp. 80–108). Cambridge University Press.
30. Sohn, H., & Lee, S.-W. (2019). Causal Impact of Having a College Degree on Women’s Fertility: Evidence From Regression Kink Designs. Demography, 56(3), 969–990. <https://doi.org/10.1007/s13524-019-00771-9>
31. Choi, S. (2015). When everyone goes to college: The causal effect of college expansion on earnings. Social Science Research, 50, 229–245. <https://doi.org/10.1016/j.ssresearch.2014.11.014>
32. Ahn, M.-S. (2011). The statistics of changes in higher education: From 5.31 educational policy reform, 1995 to 2010. Korea Research Institute of Higher Education. (In Korean)
33. Kim, Y.-B., Yang, S.-K., & Park, S.-H. (2012). An analysis of private educational expenditure transitions and trends: Analysis of the “Household Survey” data. *The Journal of Korean Education*, *39*(1), 261–284.
34. Presidential Decree No. 13875. (1993). University student quota decree [대학학생정원령]. Partial amendment effective April 2, 1993.
35. Presidential Decree No. 15665. (1998). University student quota decree [대학학생정원령]. Effective from March 1, 1998. Other laws repealed February 24, 1998.
